# Metabolic Fingerprinting of Blood and Urine of Dairy Cows Affected by Bovine Leukemia Virus: A Mass Spectrometry Approach

**DOI:** 10.3390/metabo14110624

**Published:** 2024-11-14

**Authors:** Dawid Tobolski, Grzegorz Zwierzchowski, Roman Wójcik, Klevis Haxhiaj, David S. Wishart, Burim N. Ametaj

**Affiliations:** 1 Department of Large Animal Diseases and Clinic, Institute of Veterinary Medicine, Warsaw University of Life Sciences, 02-787 Warsaw, Poland; dawid_tobolski@sggw.edu.pl; 2 Department of Agricultural, Food and Nutritional Science, University of Alberta, Edmonton, AB T6G 2P5, Canada; grzegorz.zwierzchowski@uwm.edu.pl (G.Z.); haxhiaj@ualberta.ca (K.H.); 3 Faculty of Biology and Biotechnology, University of Warmia and Mazury, 1a Oczapowskiego Str., 10-719 Olsztyn, Poland; 4 Faculty of Veterinary Medicine, University of Warmia and Mazury, 1a Oczapowskiego Str., 10-719 Olsztyn, Poland; brandy@uwm.edu.pl; 5 Departments of Biological and Computer Sciences, University of Alberta, Edmonton, AB T6G 2P5, Canada; dwishart@ualberta.ca

**Keywords:** dairy cow, bovine leukemia virus, metabolomics, serum, urine, DI/LC-MS/M

## Abstract

Objectives: This study investigated metabolic changes associated with bovine leukemia virus (BLV) infection in dairy cows, focusing on pre-parturition alterations. Methods: Metabolite identification in serum and urine samples was performed using a targeted metabolomics method, employing the TMIC Prime kit in combination with flow injection analysis and liquid chromatography–tandem mass spectrometry. Results: Of 145 cows examined, 42 (28.9%) were BLV-seropositive. Around 38% of infected cows showed high somatic cell counts indicative of subclinical mastitis, with 15 experiencing additional health issues such as ketosis, milk fever, and lameness. Despite these conditions, no significant differences in milk yield or composition were observed between the infected and control groups. Metabolomic analysis conducted at −8 and −4 weeks prepartum revealed significant metabolic differences between BLV-infected and healthy cows. At −8 weeks, 30 serum metabolites were altered, including sphingomyelins, lysophosphatidylcholines, amino acids, and acylcarnitines, suggesting disruptions in membrane integrity, energy metabolism, and immune function indicative of early neoplastic transformations. By −4 weeks, the number of altered metabolites decreased to 17, continuing to reflect metabolic disruptions in cows with leukemia. Multivariate analysis highlighted distinct metabolic profiles between infected and control cows, identifying key discriminating metabolites such as choline, aspartic acid, phenylalanine, and arginine. Urine metabolomics revealed significant prepartum shifts in metabolites related to glucose, asymmetric dimethylarginine, and pyruvic acid, among others. Conclusions: The research confirmed metabolomics’ efficacy in defining a BLV infection metabolic profile, elucidating leukosis-associated metabolic disruptions. This approach facilitates the identification of BLV-infected cows and enhances understanding of infection pathophysiology, providing a foundation for advanced management and intervention strategies in dairy herds. The study underscores the profound impact of leukosis on metabolic processes and highlights urine metabolomics’ utility in non-invasively detecting BLV infection, offering the potential for improved herd health management.

## 1. Introduction

Bovine leukemia virus (BLV), the causative pathogen of bovine leukosis, exhibits a high prevalence in North American dairy herds, with studies reporting herd prevalence rates approaching 90% [1,2]. This retrovirus not only precipitates malignant lymphosarcoma but also incurs notable economic losses, akin to the genomic characteristics it shares with the human T-cell leukemia virus type 1 (HTLV-1). BLV instigates enzootic bovine leukosis (EBL), the foremost neoplastic disease afflicting cattle. While a majority of BLV-infected cattle remain asymptomatic, thus promoting high rates of viral shedding, approximately 30% manifest persistent lymphocytosis, and a minority of less than 5% exhibit overt clinical manifestations of EBL [3].

The implications of BLV infection extend to decreased milk yield, diminished longevity of dairy cattle, compromised vaccine efficacy, and the potential development of EBL. Owing to the retroviral nature of the BLV, which guarantees permanent viral persistence once infection occurs [4], managing its prevalence necessitates the strategic culling of infected cows from herds. A notable national survey within North America revealed that 40% of dairy and 31% of beef herds in Manitoba, Canada, harbored BLV-seropositive animals. Similarly, in Wisconsin, USA, the prevalence of herds with at least one BLV-positive blood sample stood at 83% [5]. Although over 20 countries, predominantly in Europe, have successfully eradicated EBL through rigorous control and eradication initiatives [6], the BLV continues to persist across several regions, with some countries reporting an escalation in infection rates [7]. This widespread prevalence underscores the need for comprehensive measures to mitigate BLV infection and its consequential impacts on the global cattle industry.

Transmission of the BLV primarily occurs through the exchange of infected lymphocytes, notably via blood-to-blood contact, from mother to offspring, and potentially through the ingestion of infected milk or the use of contaminated equipment. Recognized pathways for transmission include the use of contaminated needles, dehorning instruments, equipment used for rectal examinations, and vector transmission by insects. Additionally, direct contact with mucosal surfaces, consumption of unpasteurized waste milk, and proximate interactions among cattle serve as viable avenues for the BLV to spread. To mitigate the risk of BLV transmission, implementing rigorous biosecurity protocols to restrict the spread through communal equipment and close contact between animals is crucial [1,8,9].

Intriguingly, research by Buehring et al. [10] identified BLV DNA in the buffy coat of human blood samples in 38% of participants from a cohort of 95 individuals, suggesting potential zoonotic dimensions of the BLV. Similarly, Szczotka and Kuźmak [11] reported the presence of the BLV in the milk lymphocytes of infected bovines. These observations underscore the necessity for more comprehensive public health studies to ascertain the risk of BLV transmission to humans, especially through dairy consumption.

Despite prevalent infection rates within cattle populations, a mere 5% of BLV-infected cows manifest clinical signs such as lymphosarcoma, typically after an extensive asymptomatic latency period ranging from 2 to 8 years [12]. Although most infections remain subclinical, they nonetheless cause economic losses, including diminished milk output, heightened rates of culling, and the emergence of secondary health complications. Many carriers of the BLV may never exhibit overt signs of the disease within their productive lifespan, attributed to the delayed onset of leukemia post-infection. These subclinical carriers further facilitate the virus’s spread within herds. Presently, there are no available treatments to completely eliminate BLV infection. Therefore, prioritizing advancements in early detection methods and investigating the metabolic pathways influenced by EBL becomes imperative. Enhancing our understanding in these areas is essential for curbing transmission and devising effective management strategies for affected herds [12].

Traditional diagnostic approaches, such as ELISA, are adept at identifying antibodies subsequent to BLV infection, yet they are constrained by the latency of seroconversion, which may span several weeks [13]. Direct detection methods like PCR, capable of pinpointing proviral DNA within host cells, often falter in sensitivity, particularly among asymptomatic cattle harboring minimal viral loads [14]. This diagnostic gap underscores the imperative for pioneering non-invasive techniques capable of early BLV identification prior to seroconversion. “Omics” technologies, with a spotlight on metabolomics, are emerging as frontiers in this regard. Metabolomics, through the comprehensive analysis of small molecules in blood or urine, can unveil characteristic metabolic disruptions in BLV-infected animals, signaling disease presence through subtle molecular shifts. This approach signals a leap towards more prompt and precise detection methodologies, facilitating immediate intervention and effective disease management [15].

Given the absence of a viable vaccine against the BLV [3], the emphasis on detection and early diagnosis becomes paramount in curtailing its transmission and mitigating economic fallout. Metabolomics, by cataloging the intricate variety of small-molecule metabolites in biological specimens under distinct conditions, renders a detailed snapshot of an organism’s physiological state. Leveraging sophisticated analytical tools, such as mass spectrometry, metabolomics defines broad metabolic alterations indicative of disease emergence or progression [16]. Employing multivariate statistical methods to contrast metabolic signatures between unaffected and BLV-afflicted cohorts, metabolomics identifies unique biomarkers. These minor but significant shifts in metabolic pathways have the potential to forewarn forthcoming disease manifestations, thus enabling pre-emptive measures. With its profound capacity to reveal early biomarkers of disease before clinical signs surface, metabolomics helps in very early detection, leading towards precision medicine and targeted disease management strategies [15].

We hypothesized that metabolomic profiling of serum and urine samples from clinically asymptomatic BLV-infected cows will reveal distinct metabolic biomarkers and alterations that can facilitate the early diagnosis of BLV infection. Furthermore, these metabolic changes can elucidate the underlying pathomechanisms of leukemia in cattle, differentiating infected from healthy individuals based on their metabolic profiles.

Therefore, the objectives of our study were: (1) to determine the capability of metabolomic profiling through DI/LC-MS/MS to identify unique biomarkers in the serum and urine of BLV-infected, asymptomatic dairy cows compared to healthy controls; (2) to analyze the metabolic shifts occurring in asymptomatic BLV-infected cows to better understand the metabolic response of the host to the viral infection and the pathomechanisms leading to leukemia; and (3) to utilize multivariate statistical analysis to compare the metabolic profiles of BLV-infected cows with those of age-matched healthy controls, aiming to highlight the metabolic disturbances indicative of BLV infection.

## 2. Materials and Methods

### 2.1. Animals, Diets, and Blood and Urine Samples

In this study, we implemented a nested case-control design, involving 145 multiparous Holstein cows from a commercial dairy operation in Alberta, Canada, focusing on those beyond their first lactation to align with our research criteria, particularly targeting the dry-off period for sample collection. This decision to exclude primiparous cows was made to ensure the consistency and specificity of our data collection. We adhered to stringent animal care protocols, approved by the University of Alberta Animal Care and Use Committee for Livestock (AUP00003216), underscoring our commitment to ethical research practices.

The cows included in the study were selected based on their expected calving dates. Sampling was strictly timed to coincide with significant phases of the dry-off period, specifically at −8 wks (55–58 days) and −4 wks (27–30 days) before the anticipated parturition. To maintain the integrity of the study and focus on the metabolic effects associated with EBL, we excluded any cows presenting with conditions like mastitis, metritis, retained placenta, laminitis, displaced abomasum, milk fever, or ketosis, either prior to or during the calving period. This exclusion was important to clearly define the metabolic alterations attributable to EBL, effectively eliminating potential confounders. The health evaluations for these criteria were conducted by a veterinary professional and were further supported by detailed health and medical records from the dairy farm’s database, ensuring a rigorous and precise selection process for our study cohort.

From the initial cohort, 30 cows met our selection criteria and were subsequently divided into two groups. Fifteen cows, identified as clinically healthy and free of the BLV, formed the control group (CON), while the other 15, diagnosed with a BLV-positive status, comprised the leukosis (LEU) group. All cows with leukosis were confirmed BLV-positive through serological testing. Cows in the BLV-infected group exhibited visible tumors under the skin, particularly around the base of the tail, legs, and other joints. Notably, during the postpartum observation period, cows in the CON group exhibited no clinical signs, underscoring the asymptomatic nature of the disease in its early stages. This aspect of the study was particularly significant, highlighting the potential for early detection and management of EBL in dairy herds.

Blood samples were drawn from the coccygeal vein, a method chosen to minimize animal distress while adhering to animal welfare standards. To collect urine samples, we first ensured the cleanliness of the vulvar area with warm water and soap to remove any debris or fecal contamination. This was followed by an alcohol disinfection step to maintain aseptic conditions. The process of gentle perineal massaging was employed to encourage natural urination, aiding in the collection of uncontaminated urine samples.

Sampling was conducted during the early morning hours, specifically between 07:00 and 08:00 a.m., and prior to the morning feeding routine. For blood collection, we utilized 10 mL Vacutainer tubes (Becton Dickinson, Franklin Lakes, NJ, USA) equipped with a clot activator and a serum separator. Urine was collected in sterile 20 mL tubes to maintain sample integrity and sterility.

Immediately following collection, blood samples were placed on ice to promote coagulation, while urine samples were also transported to the laboratory on ice, using sterile 20 mL tubes to ensure sample preservation. Serum was then extracted by centrifuging the blood at 2090 RCF for 15 min using a Rotanta 460 R centrifuge (Hettich Zentrifugan, Tuttlingen, Germany). The resulting serum was carefully transferred into sterile tubes using a transfer pipette (Fisher Scientific, Toronto, ON, Canada).

To maintain the quality of the samples for accurate analysis, 200 μL aliquots of both serum and urine were stored at −80 °C until they were analyzed. This approach to sample collection and storage was critical to ensuring the reliability and validity of our subsequent analyses.

Before analysis, all samples were thawed on ice and then vortexed to ensure uniformity. The comprehensive metabolomic analyses were carried out by The Metabolomics Innovation Centre (TMIC) at the University of Alberta, employing liquid chromatography–tandem mass spectrometry (LC-MS/MS) techniques. This approach enabled precise and detailed metabolomic profiling.

For further context, the study includes Supplementary Tables S1 and S2, which provide a detailed breakdown of the feed ingredients given to the cows both pre- and post-parturition, quantified on a dry matter basis. These tables are instrumental in understanding the nutritional regimen of the cows throughout the duration of the study.

### 2.2. Metabolite Analysis Protocol

The analysis of biogenic amines (BAs), amino acids (AAs), lipids, acylcarnitines (ACs), and glucose was conducted using a designed protocol involving a 96-well filter plate. The process began with the addition of 10 μL of flow injection analysis (FIA) running buffer and liquid chromatography (LC) internal standards (ISTDs) to each well. The first well was designated as a double blank, receiving no additions. Wells two through fourteen were prepared with a mix of control and calibration samples. This included three phosphate-buffered saline (PBS) “zero-point” control samples, seven calibration curve standards, and three quality control (QC) samples.

The thawed serum and urine samples were then allocated to the subsequent wells, with each well receiving 10 μL of sample. Following this, the plate underwent an incubation period and was then subjected to drying under a nitrogen stream using a Zanntek Analytical Evaporator (Glas-Col, Terre Haute, IN, USA) for 30 min. After drying, 50 μL of a 5% phenylisothiocyanate (PITC) solution was added to each well, followed by a 20-minute incubation at room temperature. A final drying phase, lasting 90 min under nitrogen flow, completed the sample preparation process.

To extract metabolites, we added 300 μL of methanol containing 5 mM ammonium acetate to each well of the plate. The plate was then placed on a shaker and agitated at 330 rpm for 30 min. Following this, it was centrifuged at 50× *g* for 5 min using a Sorvall Evolution RC Superspeed Centrifuge (Fisher Scientific, Toronto, ON, Canada). This process facilitated the transfer of the contents into a lower 96-deep-well plate. For the analysis of amino acids (AAs) and biogenic amines (BAs), the extract was diluted in a 1:1 ratio with water, and 10 μL of this diluted sample was injected into the analytical column. In contrast, for the analysis of acylcarnitines (ACs), lipids, and glucose, 150 μL of the extract was mixed with 400 μL of FIA running buffer, and 20 μL of this mixture was used for column injection. Each step was executed with precision to maintain the accuracy and integrity of the metabolomic analysis.

### 2.3. Organic Acid Analysis Procedure

To analyze organic acids, we employed a protein precipitation method using 1.5 mL Eppendorf tubes. Each tube received a combination of an internal standard mixture (ISTD) solution (10 μL), the sample (50 μL), and ice-cold methanol (150 μL). For blanks, calibration standards, and quality control (QC) samples, we substituted methanol with a 3:1 methanol:water solution. The tubes were then vortexed thoroughly and stored at −20 °C overnight.

The following day, the samples were centrifuged at 21,000× *g* for 15 min. Post-centrifugation, 50 μL of the supernatant from each tube was carefully transferred into the wells of a 96-deep-well plate. To each well, we added 25 μL of three reagents: 3-nitrophenylhydrazine, 1-Ethyl-3-(3-dimethylaminopropyl) carbodiimide, and pyridine. This mixture was agitated at 450 rpm for 2 h at room temperature to complete the derivatization reaction.

After the reaction, we added 350 μL of double distilled water and 50 μL of methanol (MeOH) to each well, diluting and stabilizing the solution for subsequent analysis using liquid chromatography–tandem mass spectrometry (LC-MS/MS).

### 2.4. FIA/LC—MS/MS Analysis Method

In our study, the identification of metabolites in serum and urine samples was conducted using a targeted metabolomics approach, utilizing the TMIC Prime kit and flow injection analysis/liquid chromatography–tandem mass spectrometry. The analysis was performed with an Agilent 1100 series liquid chromatographic system (LC), equipped with an Agilent reversed phase Zorbax Eclipse XDB C18 column (3.0 × 100 mm, 3.5 μm particle size, 80 Å pore size) and a Phenomenex SecurityGuard C18 pre-column (4.0 × 3.0 mm). This LC system was integrated with an AB SCIEX QTRAP^®^ 4000 mass spectrometer (Sciex Canada, Concord, ON, Canada), enhancing the detection and quantification of the metabolites.

The reagents employed in this analysis included LC/MS-grade formic acid and HPLC-grade water, both procured from Fisher Scientific (Ottawa, ON, Canada). Additionally, ammonium acetate, phenylisothiocyanate (PITC), and HPLC-grade acetonitrile (ACN) were sourced from Sigma-Aldrich (St. Louis, MO, USA). The LC-MS assay workflow was managed and controlled using Analyst^®^ 1.6.2 software (Sciex Canada, Concord, ON, Canada), which was instrumental in ensuring precise and dependable data acquisition.

For the targeted analysis of amino acids (AAs) and biogenic amines (BAs), the LC system parameters were meticulously optimized. Mobile phase A was composed of 0.2% (*v*/*v*) formic acid in water, while mobile phase B contained the same concentration of formic acid in acetonitrile. A carefully designed gradient profile was implemented to enhance the separation and detection of AAs and BAs, with specific intervals set for adjusting the proportion of mobile phase B. The column oven temperature was consistently maintained at 50 °C to ensure analytical stability, and the flow rate was set at 500 μL/min. Each sample was introduced with an injection volume of 10 μL.

For the LC-MS/MS analysis of organic acids, the solvent system included 0.01% (*v*/*v*) formic acid in water (solvent A) and 0.01% (*v*/*v*) formic acid in methanol (solvent B). The operating conditions were finely tuned for optimal analysis of organic acids, setting the column oven temperature at 40 °C and regulating the flow rate at 300 μL/min. The injection volume for these samples was also maintained at 10 μL. The mass spectrometer operated in negative electrospray ionization mode, employing scheduled multiple reaction monitoring (MRM) scans to significantly enhance the detection’s specificity and sensitivity. These selected parameters and conditions were crucial in obtaining a comprehensive and accurate metabolite profile from the serum and urine samples.

### 2.5. Statistical Analysis

In this study, we conducted univariate statistical analyses using Python (v. 3.11.2, Python Software Foundation, Frederick, MA, USA, 1991) and R (v. 4.1.0, R Foundation for Statistical Computing, Vienna, Austria, 2008). We evaluated the differences in metabolite concentrations between the clinically healthy control group (CON) and the leukemia group (LEU) at two prepartum time points (−8 weeks and −4 weeks) utilizing the Mann–Whitney U test. Metabolites demonstrating a *p*-value of 0.05 or lower were deemed statistically significant. For an in-depth analysis of the metabolomics data, we employed MetaboAnalyst 4.0 (University of Alberta, Edmonton, Canada). Missing values were addressed by implementing a minimum value imputation strategy, and to achieve normalized data distributions, we applied log transformation.

Our multivariate analysis strategy encompassed principal component analysis (PCA) and partial least squares discriminant analysis (PLS-DA) to elucidate the differences in metabolic profiles between the CON and LEU groups. The robustness of the PLS-DA model was verified via a permutation test comprising 10,000 iterations. Metabolites were prioritized based on their variable importance in projection (VIP) scores, highlighting their relevance in distinguishing between the CON and LEU groups. Additionally, receiver operating characteristic (ROC) curve analysis was performed for the top five metabolites with the highest VIP scores to assess their classification performance. The diagnostic accuracy was quantified using the area under the ROC curve (AUC).

To graphically illustrate the differences in metabolite concentrations between the CON and LEU groups, we utilized volcano plots, which displayed fold changes on the *x*-axis and statistical significance as −log10 *p*-value on the *y*-axis. Venn diagrams were employed to elucidate unique and shared metabolite alterations at each time point. We also conducted pathway enrichment analysis to identify significantly impacted metabolic pathways, taking into account both metabolite enrichment and topology. Furthermore, heatmaps were used to visually depict the relative abundance of metabolites within these pathways across the different groups, providing a clear and comprehensive representation of the metabolic alterations.

## 3. Results

In this investigation of 145 dairy cows, 42 (28.9%) were serologically identified as infected by BLV. Within this cohort of BLV-infected cows, 16 (38%) of the infected population were observed to have elevated somatic cell counts (SCCs) exceeding 200,000 cells/mL of milk, indicative of subclinical mastitis. Additionally, among the BLV-positive cows, 15 exhibited further health complications: 5 presented with ketosis, 4 with milk fever, and 3 were diagnosed with lameness. Notably, a single cow with leukosis was diagnosed with two concurrent conditions, ketosis and displaced abomasum. The study’s findings did not indicate significant differences in milk yield and composition (for the first 56 d of lactation) between the cows diagnosed with leukosis (LEU) and the control group (CON), suggesting the need for further detailed analysis to ascertain the broader impacts of BLV infection on dairy production parameters.

### 3.1. Blood Metabolomic Alterations in Leukemic Cows During Dry-Off Period

**Figure 1:**
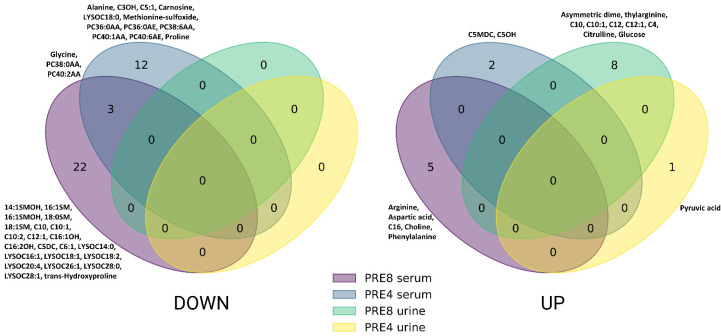
Venn diagrams illustrating significantly altered (*p* ≤ 0.05) metabolites in serum and urine samples from cows with bovine leukemia virus (BLV) infection compared to healthy controls at −8 and −4 weeks prepartum.

Utilizing univariate analysis with the Mann–Whitney U test, we observed significant differences in metabolite concentrations between the LEU and CON groups within serum samples. Specifically, at −8 wks before the expected parturition, differential analysis revealed distinct variations in 30 metabolites (*p* < 0.05) in the serum, effectively differentiating the two groups. This difference decreased to 17 metabolites (*p* < 0.05) at −4 wks prepartum, illustrating the dynamic nature of metabolic shifts as the calving period approached (Figure 1, Table 1 and Table 2).

This figure presents two Venn diagrams showing the distribution of down-regulated (LEFT) and up-regulated (RIGHT) metabolites in BLV-infected cows versus healthy controls. The diagrams compare metabolite changes in serum and urine at two critical time points: 8 and 4 weeks before expected calving. Each circle represents a specific sample type and timepoint, with numbers indicating unique or shared metabolites. Key altered metabolites are listed beside their respective circles.

**Figure 2:**
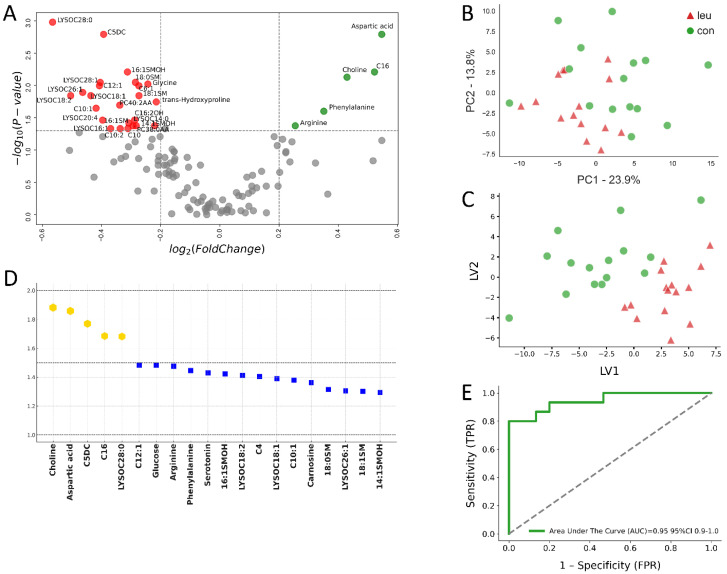
Serum metabolomic analysis of cows with bovine leukemia virus (BLV) infection compared to healthy controls at −8 weeks prepartum. This multi-panel figure presents a comprehensive metabolomic analysis of serum samples from 15 cows with leukemia (LEU) and 15 clinically healthy controls (CON) at −8 weeks before expected calving. (**A**) The volcano plot illustrates differentially expressed metabolites, with significant changes (*p* ≤ 0.05) highlighted in red (down-regulated) and green (up-regulated) for fold changes Log_2_(FC) ≤ −0.2 or ≥0.2, respectively. Metabolites with no significant changes are shown in grey. Key altered metabolites are labeled. (**B**) Principal component analysis (PCA) and (**C**) partial least squares discriminant analysis (PLS-DA) demonstrate clear separation between the LEU and CON groups, indicating distinct metabolic profiles. (**D**) Variables ranked by variable importance in projection (VIP) scores identify the most discriminative metabolites, with the top five highlighted in gold and an additional 15 metabolites indicated by blue squares. (**E**) The receiver operating characteristic (ROC) curve for the top five metabolites shows excellent diagnostic potential with an area under the curve (AUC) of 0.95 (95% CI: 0.9–1.0). Together, these analyses reveal significant metabolic alterations in BLV-infected cows during the dry-off period, potentially offering early biomarkers for disease detection and monitoring.

**Figure 3:**
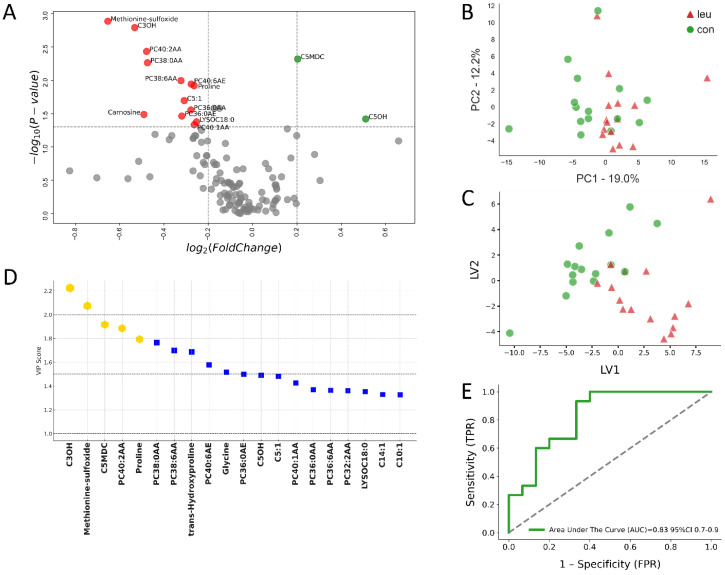
Serum metabolomic analysis of cows with bovine leukemia virus (BLV) infection compared to healthy controls at −4 weeks prepartum. This multi-panel figure presents a comprehensive metabolomic analysis of serum samples from 15 cows with leukemia (LEU) and 15 clinically healthy controls (CON) at −4 weeks before expected calving. (**A**) The volcano plot illustrates differentially expressed metabolites, with significant changes (*p* ≤ 0.05) highlighted in red (down-regulated) and green (up-regulated) for fold changes Log_2_(FC) ≤ −0.2 or ≥0.2, respectively, whereas highlighted in grey are metabolites with no significant change. Key altered metabolites are labeled. (**B**) Principal component analysis (PCA) and (**C**) partial least squares discriminant analysis (PLS-DA) show separation between the LEU and CON groups, indicating distinct metabolic profiles closer to parturition. (**D**) Variables ranked by variable importance in projection (VIP) scores identify the most discriminative metabolites, with the top five highlighted in gold and an additional 15 metabolites indicated by blue squares. (**E**) The receiver operating characteristic (ROC) curve for the top five metabolites demonstrates good diagnostic potential with an area under the curve (AUC) of 0.83 (95% CI: 0.7–0.9). These analyses reveal ongoing metabolic alterations in BLV-infected cows as they approach calving, providing insights into disease progression and potential biomarkers for monitoring.

Further exploration through volcano plot analysis revealed crucial metabolic alterations in the serum of LEU cows during the prepartum phase. At the −8 wks mark, significant reductions in specific sphingomyelins (SMs) and phosphatidylcholines (PCs), such as 14:1SMOH and 16:1SM, were recorded, alongside elevations in metabolites such as arginine and phenylalanine (*p* < 0.05) (Figure 2A). Approaching parturition, at −4 wks prepartum, the volcano plot depicted a reduced array of significant metabolic differences between the LEU and CON groups. Noteworthy changes included decreased concentrations of metabolites such as carnosine and methionine-sulfoxide, coupled with increases in C5MDC and C5OH (*p* < 0.05), highlighting the ongoing metabolic adaptation and response in cows affected by leukosis (Figure 3A).

In our multivariate analysis of serum samples, a pronounced differentiation in the metabolic profiles of the LEU and CON groups was evident, as depicted in Figure 2A–E. At −8 wks prior to parturition, the application of PLS-DA yielded a high degree of predictive accuracy. Critical metabolites, namely choline, aspartic acid, phenylalanine (Phe), and arginine (Arg), emerged as significant discriminators, marked by their heightened variable importance in projection (VIP) scores, effectively differentiating the LEU group from the CON group (as shown in Figure 2D).

The receiver operating characteristic (ROC) curve analysis for serum, zeroing in on the five metabolites with the highest VIP scores—choline, aspartic acid, C5DC, C16, and LYSOC28:0—demonstrated superior classification capability. The area under the curve (AUC) for this assessment reached 0.95 (95% confidence interval: 0.90–1.0, *p* < 0.001), highlighting the reliability of these metabolites in accurately distinguishing between the LEU and CON cows (illustrated in Figure 2E).

Progressing to the −4 week prepartum interval, our multivariate analysis persisted in defining a clear division between the metabolic signatures of the LEU and CON cows, as illustrated in Figure 3A–E. The PLS-DA retained its elevated predictive accuracy, with VIP analysis pinpointing C3OH, methionine-sulfoxide, C5MDC, PC40:2AA, and proline (Pro) as the foremost discriminative metabolites for this stage, affirming the robustness of metabolomic profiling in discerning between health statuses among dairy cows.

The ROC curve analysis conducted at −4 wks prepartum revealed commendable predictive accuracy, with an AUC value of 0.83, employing these principal metabolites for differentiation between the LEU and CON groups, as shown in Figure 3E. The variations in these metabolites signal underlying metabolic disturbances in cows affected by leukosis, highlighting their potential as biomarkers for early detection and diagnosis.

### 3.2. Urinary Metabolomic Alterations in Leukosis Cows During the Dry-Off Period

The analysis of urine samples revealed significant metabolic changes in cows affected by leukemia, identifying eight metabolites with significant differences at −8 wks before parturition (*p* < 0.05) and a single metabolite at −4 wks before parturition (*p* < 0.05). These findings underscore the metabolic impact of leukosis on these animals, as detailed in Table 3 and Table 4, alongside Figure 1.

Specifically, in the urine samples from cows diagnosed with leukosis, our volcano plot analysis highlighted significant shifts in metabolites before parturition. Eight weeks prior to parturition, we detected significant increases in concentrations of specific metabolites, including glucose, asymmetric dimethylarginine, and citrulline, as well as short- and medium-chain fatty acids (C4, C10, C10:1, C12, and C12:1) (*p* < 0.05). Closer to parturition, at −4 wks before, a significant rise in pyruvic acid (*p* < 0.05) was the only notable change, pointing to distinct metabolic disruptions as calving approaches (Figure 1).

**Figure 4:**
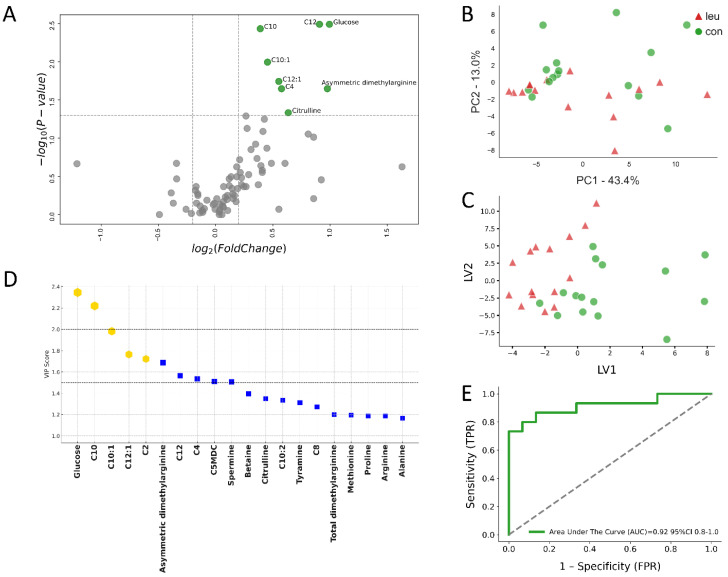
Urinary metabolomic analysis of cows with bovine leukemia virus (BLV) infection compared to healthy controls at −8 weeks prepartum. This multi-panel figure presents a comprehensive metabolomic analysis of urine samples from 15 cows with leukemia (LEU) and 15 clinically healthy controls (CON) at −8 weeks before expected calving. (**A**) The volcano plot illustrates differentially expressed metabolites, with significant changes (*p* ≤ 0.05) highlighted in green (up-regulated) for fold changes Log_2_(FC) ≥ 0.2. Metabolites with no significant changes are shown in grey. Key altered metabolites are labeled. (**B**) Principal component analysis (PCA) and (**C**) partial least squares discriminant analysis (PLS-DA) demonstrate clear separation between the LEU and CON groups, indicating distinct urinary metabolic profiles. (**D**) Variables ranked by variable importance in projection (VIP) scores identify the most discriminative metabolites, with the top five highlighted in gold and an additional 15 metabolites indicated by blue squares. (**E**) The receiver operating characteristic (ROC) curve for the top five metabolites shows excellent diagnostic potential with an area under the curve (AUC) of 0.92 (95% CI: 0.8–1.0). These analyses reveal significant urinary metabolic alterations in BLV-infected cows during the early dry-off period, potentially offering non-invasive biomarkers for early disease detection and monitoring.

**Figure 5:**
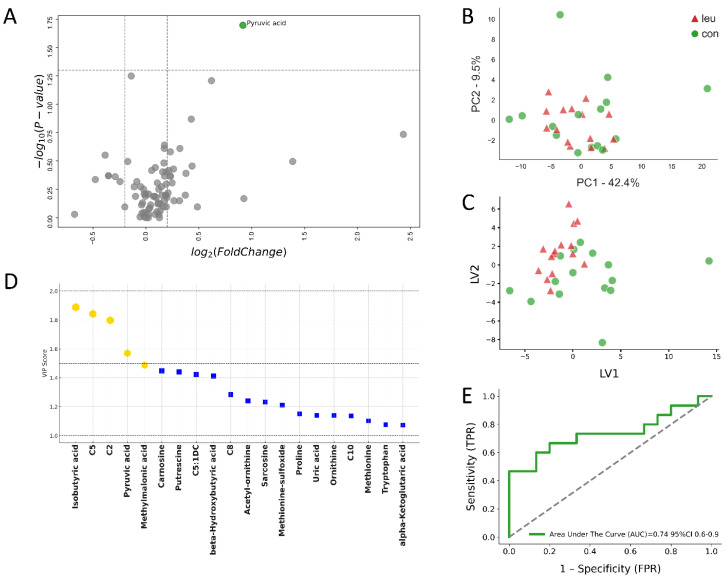
Urinary metabolomic analysis of cows with bovine leukemia virus (BLV) infection compared to healthy controls at −4 weeks prepartum.This multi-panel figure presents a comprehensive metabolomic analysis of urine samples from 15 cows with leukemia (LEU) and 15 clinically healthy controls (CON) at −4 weeks before expected calving. (**A**) The volcano plot illustrates differentially expressed metabolites, with pyruvic acid standing out as significantly up-regulated (*p* ≤ 0.05, Log_2_(FC) ≥ 0.2) in LEU cows. Metabolites with no significant changes are shown in grey. (**B**) Principal component analysis (PCA) and (**C**) partial least squares discriminant analysis (PLS-DA) show some separation between the LEU and CON groups, indicating distinct urinary metabolic profiles closer to parturition. (**D**) Variables ranked by variable importance in projection (VIP) scores identify the most discriminative metabolites, with the top five highlighted in gold and an additional 15 metabolites indicated by blue squares. (**E**) The receiver operating characteristic (ROC) curve for the top five metabolites demonstrates moderate diagnostic potential with an area under the curve (AUC) of 0.74 (95% CI: 0.6–0.9). These analyses reveal ongoing urinary metabolic alterations in BLV-infected cows as they approach calving, albeit with less pronounced differences compared to the 8-week prepartum timepoint. This suggests a dynamic metabolic response to BLV infection throughout the dry-off period, potentially offering insights into disease progression and metabolic adaptation.

Furthermore, our multivariate analysis of urinary metabolites revealed a clear differentiation between the metabolic profiles of cows with leukosis compared to those in the control group (CON), as depicted in Figure 4 and Figure 5. This differentiation was particularly evident at −8 wks prepartum, with the PLS-DA demonstrating a high level of predictive accuracy (Figure 4C). Notably, metabolites such as glucose and C10 were identified as key factors in distinguishing between the LEU-affected cows and the CON group, evidenced by their high variable importance in projection (VIP) scores (Figure 4D). This highlights their significance in understanding the metabolic impact of leukosis on cows.

The ROC curve analysis of urine samples collected −8 wks before parturition, focusing on metabolites such as glucose, C10, C10:1, C12:1, and C2—those with the highest VIP scores—showcased excellent classification performance. The AUC for this analysis reached an impressive accuracy of 0.92, indicating a high level of precision in using these urinary metabolites to differentiate between cows affected by leukosis (LEU) and those in the healthy control group, as detailed in Figure 4E.

In the analysis performed −4 wks before parturition, the metabolic profiles in urine samples further delineated the distinction between the LEU and CON groups, as evidenced in Figure 5A–E. At this stage, metabolites like pyruvic acid and isobutyric acid were identified as significant contributors to the metabolic discrepancies observed, as highlighted in Figure 5D.

The ROC analysis at −4 wks before parturition presented an AUC of 0.74. Although this figure represents a somewhat lesser degree of classification accuracy compared to the analysis at eight weeks prepartum, it still reflects a noteworthy capability for discerning between the groups, as depicted in Figure 5E. The consistent detection of metabolic changes at both the −8-week and −4-week marks before parturition underscores the utility of urine metabolomics as a potential diagnostic approach for the early identification of leukosis in cattle. This method promises a non-invasive and efficient way to identify animals at risk, enabling early intervention and informed management decisions.

## 4. Discussion

To our knowledge, this study represents the first comprehensive analysis of the metabolomic fingerprint associated with bovine leukemia virus (BLV) infection in dairy cows. Metabolic fingerprinting provides a precise snapshot of the host’s metabolic state at the moment of sampling. This research is pioneering in its examination of the metabolic alterations observed in the blood and urine of dairy cows that have been serologically confirmed to carry BLV infection. The sampling was intentionally performed at two critical time points: two months and one month prior to parturition. These time points were selected to gain insights into the impact of BLV infection on the physiological processes of the host during late pregnancy, a critical phase for the well-being of the calf and the initiation of lactation.

There is evidence that BLV infection negatively affects the milk yield and overall health of cows, making them more susceptible to other diseases around parturition. For instance, previous studies have identified an increased risk of both subclinical and clinical mastitis in dairy cows with a high proviral load [17,18]. Indeed, the analysis revealed that 38% of the cows (n = 42) diagnosed with leukosis also had subclinical mastitis. Additionally, 35.7% of the leukotic cows (15 out of the 42 cows with leukosis, from the total study group of 145) presented with one or multiple periparturient diseases, such as ketosis, milk fever, lameness, or displaced abomasum, in contrast to the healthy control group cows, which exhibited no such diseases.

Our study aims to further elucidate the metabolic repercussions of BLV infection by providing a detailed discussion of the key metabolic findings, focusing on the specific alterations induced by the infection in the serum and urine of dairy cows at two significant points during the dry-off period.

### 4.1. Blood Alterations in BLV-Infected Cows During the Dry-Off Period

One of the noteworthy observations from our study was a significant change in lipid metabolism, highlighted by a noticeable decrease in lipid molecules: eight molecular species of LysoPCs, two species of PCs, and five species of SMs in cows infected with BLV observed −8 weeks prior to parturition. Furthermore, −4 weeks before parturition, we detected a decrease in seven molecular species of PCs and one LysoPC in BLV-infected cows. These findings align with research in human acute myeloid leukemia (AML) patients, which documented lowered concentrations of LysoPCs, PCs, and SMs in individuals with the leukemia virus [19].

In the context of tumor cells, including those infected by BLV, there is an increased demand for PCs, driven by rapid cell growth and the necessity for membrane biosynthesis. The synthesis of PCs occurs via the cytidine-diphosphate (CDP)-choline pathway, where choline is converted into phosphocholine and then combined with diacylglycerol [20]. Conversely, LysoPCs are generated from the hydrolysis of PCs, a reaction catalyzed by phospholipase A2 (PLA2) [21]. These molecules play a crucial role in maintaining membrane structure and fluidity, fundamental for a range of cellular functions. Additionally, they act as signaling molecules, potentially involved in processes such as inflammation, cell migration, and proliferation.

BLV infection specifically targets B cells in dairy cows, leading to leukosis characterized by the abnormal proliferation of these infected B cells as well as immune cells [22]. This increased proliferation necessitates additional resources, particularly for membrane synthesis, to support the growing population of cells [23]. The resulting demand for increased membrane synthesis in BLV-infected B cells contributes to the altered metabolism of LysoPCs and PCs. As LysoPCs are crucial components of cell membranes, their metabolism is significantly affected by the increased need for new cell membranes.

In the case of sphingomyelins, which are synthesized from ceramide and phosphocholine, their turnover is vital in tumor cells for maintaining membrane structure and facilitating the production of bioactive lipids like ceramide [24]. Sphingomyelins are integral to signal transduction mechanisms and have a role in regulating processes such as apoptosis, often disrupted in cancerous cells [25]. This underscores the intricate relationship between lipid metabolism and the biological functions of B cells in the progression of BLV-induced leukosis in dairy cows.

In B cells infected by BLV, as in other types of leukemic cells, there is a distinctive shift in lipid metabolism towards increased uptake and utilization of lipids such as PCs, LysoPCs, and SMs. This shift is necessary to accommodate rapid cell division and the synthesis of new cellular membranes [26]. Such an escalated lipid demand may result in diminished levels of these lipids in circulation. The immune system’s response to BLV infection, characterized by the activation and proliferation of various immune cells, further amplifies this lipid consumption for membrane synthesis and signaling, exacerbating the decline in their serum concentrations [27].

The study observed a decrease in choline levels in serum among BLV-infected dairy cows. Choline is a vital element of phosphatidylcholine, and its depletion might reflect intensified demands for membrane synthesis and repair, driven by elevated cell turnover or immune activation [28]. Beyond its structural role, choline acts as a methyl donor in several biochemical pathways, including those involved in DNA and histone methylation, essential for regulating gene expression [29]. Additionally, choline plays a role in lipid transport and metabolism, notably in the formation of lipoproteins [30]. A decrease in choline could signify adjustments in lipid management, likely due to the metabolic challenges posed by BLV infection or the host’s immune reaction.

Our study identified significant changes in the amino acid profiles of dairy cows, distinguishing the control group from those infected with BLV. Specifically, there was an increase in the concentrations of arginine, aspartic acid (aspartate), phenylalanine, and choline in the serum of BLV-infected cows at −8 wks before parturition, followed by a decrease in alanine, glycine, proline, methionine-sulfoxide, and carnosine at −4 wks prepartum.

Arginine and its metabolic pathways have been linked to tumorigenesis [31]. Some tumors depend on external sources of arginine for growth, classifying them as either completely or partially arginine-dependent (arguably auxotrophic or semi-auxotrophic) [32]. The presence of enzymes involved in arginine biosynthesis, like argininosuccinate synthase (ASS1) and argininosuccinate lyase (ASL), plays a crucial role in determining a tumor’s arginine auxotrophy [33]. In the context of our research, inducing arginine deficiency through NEI-01 led to the death of ASS1-deficient acute myeloid leukemia (AML) cells by triggering sub-G1 cell cycle arrest and apoptosis. Beyond promoting apoptosis, arginine deprivation is known to exhibit anti-angiogenic properties and inhibit de novo protein synthesis [34], highlighting the complex role of amino acid metabolism in the pathophysiology of BLV infection and its associated cellular changes.

Aspartate is integral to the biosynthesis of pyrimidines, one of the two categories of nitrogenous bases found in nucleic acids, with the other category being purines [35]. Pyrimidines constitute essential elements of both DNA and RNA, playing vital roles in numerous cellular operations such as cell division and protein synthesis. Research by Van Gastel et al. [36] highlighted that aspartate levels in the bone marrow plasma of mice with acute myeloid leukemia (AML) were significantly elevated—up to 70 times higher than in peripheral blood. This finding underscores the importance of aspartate for leukemic cells, given its critical role in pyrimidine synthesis. Aspartate also participates in transamination reactions that are fundamental for both the creation and degradation of amino acids. The observed increase in aspartate levels in the serum of BLV-infected cows within our study could reflect an up-regulation of amino acid metabolism, likely driven by heightened protein turnover as a response to the infection [37]. This suggests that alterations in aspartate metabolism could be a marker of underlying metabolic shifts associated with BLV infection and its impact on cellular functions.

Phenylalanine, identified as elevated in the serum of cows with a BLV infection, serves as a foundational protein building block, essential for synthesizing new proteins. Beyond its role in protein construction, phenylalanine is a precursor to several critical biomolecules, including tyrosine. Tyrosine, in turn, is vital for synthesizing neurotransmitters such as dopamine, norepinephrine, and epinephrine, as well as the pigment melanin [35]. Although not a direct energy source, phenylalanine can contribute to glucose production through gluconeogenesis under specific conditions, such as prolonged fasting or in certain metabolic disorders [38].

Research has shown that dietary phenylalanine restriction to 0.08% or lower can significantly reduce the growth of intraperitoneal leukemia tumors in mice, an effect not observed with restrictions on other amino acids like isoleucine, leucine, cysteine-methionine, or overall protein [39]. Furthermore, the lifespan of mice with L1210 leukemia or P288 leukemia was extended when phenylalanine intake was restricted to 0.08% of their diet. This suggests that dietary intervention might enhance the host’s defense mechanisms rather than directly inhibiting tumor growth [40].

The increased serum concentrations of phenylalanine observed in cows affected by BLV could have adverse implications, potentially favoring the proliferation of BLV-infected cells. This highlights the complex interplay between diet, metabolism, and disease progression, underscoring the potential of nutritional strategies in influencing the course of leukosis conditions.

The lowering of serum concentrations of glycine, proline, trans-hydroxyproline, alanine, and methionine-sulfoxide observed in dairy cows during the prepartum period (−8 and −4 wks) suggests potential metabolic and physiological adaptations, particularly within the milieu of leukemic cell activity. Glycine, known for its role in glutathione synthesis—a critical antioxidant—and in purine production, necessary for nucleic acid synthesis, has far-reaching implications [41]. Decreased glycine levels may result in diminished antioxidant defense mechanisms, elevating oxidative stress, which could compromise health and immune functionality. In the context of leukemic cells, characterized by high rates of proliferation and purine demand, a glycine shortfall could restrict their growth capacity [42].

In contrast, recent research by Verstraete et al. [43] highlights that, while the majority of cells derive serine and glycine from extracellular sources, about 30% of cancers, including T-cell acute leukemia and acute myeloid leukemia, engage the de novo synthesis pathways for serine and glycine. This adaptation supports their proliferation and survival by enabling the production of serine/glycine independently [44]. Through a branch of the glycolytic pathway, cancer cells can generate serine and glycine, which not only serve as antioxidants but also provide essential components for purine synthesis. This dual role underscores the complexity of metabolic regulation in cancer cells, where alterations in amino acid levels reflect both the demands of rapid cell division and the strategies employed by cells to meet these demands.

Proline and its derivative, trans-hydroxyproline, play important roles in collagen synthesis and maintaining connective tissue integrity [45]. Decreased levels may impair tissue repair and compromise the structural integrity of the cow, including mammary tissue health, indirectly affecting the environment for leukosis cell proliferation. Furthermore, proline-rich antimicrobial peptides, essential for the innate immune response and containing high levels of L-proline residues (up to 50%), may be compromised with decreased serum proline, potentially weakening the cow’s initial defense against infections [46,47].

Alanine is crucial for glucose metabolism via gluconeogenesis, serving as an important energy source for tissues. Lowered alanine levels suggest a shift in energy metabolism, possibly due to increased energy consumption by leukemic cells or compromised liver function, affecting the overall metabolic balance [48]. Methionine sulfoxide, the oxidized form of the essential amino acid methionine, is key for methylation and antioxidant defenses, leading to cysteine and glutathione synthesis. A decline in its levels may reflect changes in redox balance and methylation activity, influencing DNA regulation and potentially impacting epigenetic control in cancer cells [49,50].

The observed reduction in blood amino acids suggests a notable metabolic reorganization in the cow, possibly driven by the leukemic cells’ heightened demands for energy and biosynthetic precursors. Leukemic cells are characterized by a distinctive metabolic adaptation, the Warburg effect, which involves elevated glucose consumption and lactate secretion, even under aerobic conditions, potentially diverting resources from the cow’s normal physiological functions [51]. This shift may hint at disrupted protein synthesis and degradation rates. During the prepartum phase, cows experience profound physiological adjustments in preparation for lactation, a process that could be further complicated by leukosis [4]. Depleted essential amino acids like glycine, important for immune system efficacy, compromise the cow’s defensive capacity against leukosis and other infections [52]. The diminished presence of amino acids vital for antioxidant defense points to elevated oxidative stress, a hallmark of cancer that influences both tumor development and host well-being [53]. Thus, the decrease in these specific amino acids during the prepartum period could reflect a redirection of metabolic resources in favor of leukosis cell proliferation, accompanied by increased oxidative stress and alterations in protein metabolism, with significant consequences for the health of the host and the dynamics of the leukosis cells [54].

Our findings indicate a reduction in serum carnosine levels in cows diagnosed with leukosis at −8 and −4 weeks prepartum. Carnosine, a dipeptide made of β-alanine and L-histidine, is predominantly found in skeletal muscles and brain tissue, playing multiple roles, including maintaining pH balance in muscles and exhibiting anti-inflammatory, antioxidant, anti-glycation, and metal ion chelating effects [55]. Research by Nagai and Suda [56] revealed that carnosine, along with beta-alanine, has immunoregulatory functions, activating both T and B cells. Notably, Prakash et al. [57] identified carnosine’s anticancer properties, particularly against U937 cells, a promonocytic human myeloid leukemia cell line. Carnosine was shown to inhibit leukemic cell proliferation, enhancing the secretion of immunomodulatory cytokines IL-10, GM-CSF, and TNF, reducing IL-8 secretion, and up-regulating gene expression of IL-8, IL-1b, TNF, as well as the expression of immune cell surface markers CD11b, CD11c, CD86, and MHCII, underscoring its anti-proliferative effects. The observed decrease in serum carnosine in prepartum cows with leukosis could, therefore, potentially contribute to the enhanced proliferation of leukotic cells.

Our research also uncovered a marked decline in serum acylcarnitines, specifically C10, C10:1, and C12:1. Acylcarnitines, carnitine, and fatty acid derivatives play an essential role in transporting fatty acids into mitochondria for β-oxidation, a critical energy production process [58]. BLV infection may disrupt cellular metabolism, as viruses typically modify the metabolism of host cells to enhance their own replication and survival, including rerouting energy sources, modifying oxidative stress mechanisms, and impairing mitochondrial functionality [59]. These viral-induced alterations could modify fatty acid metabolism, as evidenced by the observed changes in acylcarnitine levels. Such changes may stem from a shift in energy substrate preference (from lipids to carbohydrates), variations in the expression of β-oxidation enzymes, or disruptions in fatty acid transport into mitochondria [60]. In the broader context of BLV-induced systemic infection, factors including the animal’s nutritional status, hormonal fluctuations, and the energetic requirements of mounting an immune response can further affect mitochondrial efficiency and acylcarnitine oxidation [61].

### 4.2. Urinary Metabolite Alterations in BLV-Infected Cows During the Dry-Off Period

This study revealed an increase in various acylcarnitines, amino acid derivatives, and glucose in the urine of dairy cows serologically diagnosed with BLV at −4 wks prepartum. Specifically, pyruvate and propionylcarnitine were elevated at −8 wks prepartum, while nine metabolites—including C4, C8, C10, C10:1, C12, C12:1, glucose, citrulline, and ADMA—were elevated at −4 wks prepartum. Our previous research indicated that cows with subclinical mastitis post-calving exhibited increased urinary acylcarnitine excretion at −4 wks prepartum, suggesting a link between pre-disease states and acylcarnitine excretion [62,63]. Changes in acylcarnitine urinary excretion in cows with subclinical mastitis were observed not only before disease diagnosis at −8 and −4 wks prepartum but also during and after the diagnosis week, up to +8 wks postpartum [62].

Acylcarnitines, which are fatty acids bonded to carnitine, play a crucial role in transporting long-chain fatty acids into mitochondria for β-oxidation, a key energy production process [58]. Within the mitochondria, these fatty acids are degraded to acetyl-CoA, which then participates in the citric acid (TCA) cycle to produce ATP. In conditions such as leukosis, significant metabolic alterations occur, including changes in lipid metabolism [64]. Cancer cells may increase lipolysis and fatty acid oxidation as alternate pathways for energy, potentially leading to acylcarnitine accumulation if the rate of fatty acid release exceeds their mitochondrial utilization [65].

Acylcarnitines, while not toxic by themselves, signify a metabolic disequilibrium when they accumulate [66]. Typically, the body ensures a balance between acylcarnitine production and use. However, in disease states, their buildup in tissues may serve as a protective mechanism against the detrimental effects of excess fatty acids and their partially oxidized derivatives. The excretion of acylcarnitines via urine can then act as a regulatory measure to preserve metabolic equilibrium and safeguard cells from damage by these intermediates [58]. Cancer cells often exhibit mitochondrial dysfunction, aggravating the situation by impairing the mitochondria’s capacity to efficiently carry out β-oxidation, thereby leading to acylcarnitine accumulation [64].

In the context of leukosis, despite acylcarnitines being potential energy substrates, their utilization is hampered due to metabolic alterations, such as a shift towards glycolysis (the Warburg effect) and mitochondrial inefficiency [67]. These disruptions hinder the normal processing of acylcarnitines through β-oxidation and the TCA cycle, preventing their effective conversion into energy. Consequently, this leads to their buildup and subsequent excretion, reflecting a diversion from typical energy metabolism pathways in cancer cells [68].

An analysis of urine samples from kidney cancer patients and corresponding mouse models revealed that urinary acylcarnitines increase in correlation with tumor grade. It is posited that these compounds originate directly from the tumor tissue, possessing cytotoxic and immunomodulatory properties that could favor tumor growth and persistence within the host [69]. Mass spectrometry imaging analysis in a human breast tumor xenograft model identified two acylcarnitines, palmitoylcarnitine and stearoylcarnitine, with significant overlap in hypoxic tumor regions. This finding indicates a potential impairment of the fatty acid β-oxidation process within mitochondria [70]. Given the critical role of acylcarnitines in various diseases, they hold promise as valuable biomarkers for clinical diagnosis. Investigating acylcarnitines’ function could enhance our understanding of disease mechanisms and advance the development of diagnostic and therapeutic approaches.

Our research showed that cows afflicted with BLV infection exhibited a marked rise in urinary pyruvic acid −8 wks before parturition and increased concentrations of glucose, citrulline, and ADMA −4 wks before calving. Remarkably, glucose levels in the urine of infected cows were nearly twice those of uninfected ones. These metabolic alterations hint at various underlying biological mechanisms. The surge in pyruvic acid, a crucial product of glycolysis, suggests an increase in glycolytic activity, likely due to the altered metabolic needs of BLV-infected cells. This may reflect a shift toward anaerobic metabolism, similar to the Warburg effect observed in cancer cells, to meet the heightened energy requirements of proliferating leukotic cells [71,72]. The significant glucosuria indicates a disturbance in glucose metabolism, which could signal insulin resistance or impaired glucose uptake by cells, a condition possibly exacerbated by the metabolic strain of BLV infection [73]. The elevation in citrulline levels suggests an upsurge in amino acid metabolism, likely due to increased protein synthesis and turnover in response to infection [74]. Moreover, the rise in ADMA, an inhibitor of nitric oxide synthase, points to potential vascular dysfunction, perhaps due to systemic inflammation or altered hemodynamics induced by BLV infection [75]. Collectively, these findings illustrate a profound metabolic disruption in BLV-infected dairy cows, characterized by changes in pyruvic acid, glucose, citrulline, and ADMA levels. This intricate mosaic of glucose dysregulation, altered amino acid metabolism, and potential vascular modifications offers crucial insights into BLV infection’s pathophysiology, underscoring the importance of targeted strategies for managing the health and productivity of affected cows.

## 5. Conclusions

Overall, this study investigated the metabolomic profile associated with BLV infection in dairy cows prior to parturition, with a focus on changes in blood and urine metabolites at −8 and −4 wks prepartum. Data revealed notable disruptions in lipid metabolism, specifically in LysoPCs, PCs, and SMs, mirroring patterns observed in human acute myeloid leukemia. Such metabolite alterations suggest an increased need for membrane biosynthesis in BLV-infected cells, particularly during the critical phases of late pregnancy, thereby influencing outcomes related to pregnancy and lactation. The research also highlights significant shifts in amino acid profiles, including changes in levels of arginine, aspartate, phenylalanine, and carnosine, which are crucial for processes like cell division, protein synthesis, and immune function.

Moreover, the study documented notable changes in urinary metabolites during the dry-off period in cows with BLV. A marked increase in pyruvate levels at −8 wks pre-parturition and a rise in acylcarnitines, glucose, citrulline, and ADMA at −4 wks before calving were observed. Elevated urinary pyruvate suggests heightened glycolytic activity, a likely result of BLV-induced metabolic shifts. The increase in acylcarnitines reflects a metabolic imbalance, possibly due to increased lipolysis and fatty acid oxidation, a characteristic of pathological conditions like cancer, leading to acylcarnitine buildup when fatty acid release exceeds mitochondrial utilization. Furthermore, the significant rise in urinary glucose levels, nearly double those of uninfected cows, indicates a disturbance in glucose metabolism, hinting at insulin resistance or reduced glucose uptake. These findings reveal extensive metabolic reprogramming in BLV-infected dairy cows, characterized by disrupted carbohydrate and lipid metabolism, underscoring the importance of targeted strategies for BLV management. Finally, we observed a high incidence of subclinical mastitis in 38% of the cows diagnosed with leukosis and found that 35.7% of these cows were diagnosed with other periparturient diseases such as ketosis, milk fever, lameness, or displaced abomasum. These findings underline the significant health challenges BLV-infected cows face, emphasizing the necessity for effective management and intervention strategies to improve the health and productivity of dairy herds affected by BLV.

## Figures and Tables

**Table 1:** Concentrations of serum metabolites [Mean (SD)] in clinically healthy (CON) and leukemia (LEU) cows −8 weeks prepartum as determined by DI/LC-MS/MS.

−8 Weeks Prepartum						
Metabolite, μM ^1^	CON (SD)	LEU (SD)	*p*-Value	FC	Log_2_(FC) ^2^	LEU/CON
C0	3.43 (1.01)	3.83 (0.89)	0.361	1.11	0.16	UP
C2	1.60 (0.42)	1.61 (0.51)	0.868	1.00	0.01	UP
C3	0.21 (0.05)	0.19 (0.06)	0.229	0.89	−0.16	DOWN
C3:1	0.03 (0.01)	0.03 (0.01)	0.245	0.90	−0.15	DOWN
C3-OH	0.02 (0.00)	0.02 (0.00)	0.245	1.08	0.11	UP
C4	0.08 (0.02)	0.09 (0.02)	0.068	1.16	0.21	UP
C4:1	0.02 (0.00)	0.02 (0.00)	0.158	0.89	−0.17	DOWN
C4-OH (C3-DC)	0.03 (0.01)	0.03 (0.01)	0.147	0.87	−0.21	DOWN
C5	0.07 (0.01)	0.06 (0.01)	0.590	0.94	−0.09	DOWN
C5:1	0.02 (0.00)	0.01 (0.00)	0.171	0.88	−0.18	DOWN
C5:1-DC (C6-OH)	0.02 (0.00)	0.02 (0.00)	0.263	0.90	−0.16	DOWN
C5-DC	0.01 (0.00)	0.01 (0.00)	0.002	0.76	−0.39	DOWN
C5-M-DC	0.02 (0.00)	0.02 (0.00)	0.184	0.93	−0.11	DOWN
C5-OH (C3-DC-M)	0.07 (0.02)	0.06 (0.02)	0.199	0.84	−0.25	DOWN
C6	0.05 (0.02)	0.04 (0.01)	0.135	0.83	−0.27	DOWN
C6:1	0.03 (0.01)	0.03 (0.01)	0.010	0.83	−0.27	DOWN
C8	0.01 (0.00)	0.01 (0.01)	0.507	1.04	0.06	UP
C9	0.01 (0.00)	0.01 (0.00)	0.106	0.90	−0.16	DOWN
C10	0.05 (0.01)	0.04 (0.01)	0.046	0.81	−0.31	DOWN
C10:1	0.09 (0.02)	0.07 (0.03)	0.023	0.75	−0.42	DOWN
C10:2	0.02 (0.01)	0.02 (0.01)	0.046	0.79	−0.34	DOWN
C12	0.02 (0.00)	0.02 (0.01)	0.868	1.05	0.07	UP
C12:1	0.05 (0.01)	0.04 (0.01)	0.010	0.75	−0.41	DOWN
C12-DC	0.02 (0.00)	0.01 (0.01)	0.062	0.76	−0.40	DOWN
C14	0.02 (0.01)	0.02 (0.01)	0.534	0.96	−0.06	DOWN
C14:1	0.06 (0.03)	0.05 (0.02)	0.431	0.86	−0.22	DOWN
C14:1-OH	0.01 (0.00)	0.01 (0.00)	0.068	0.85	−0.23	DOWN
C14:2	0.01 (0.00)	0.01 (0.00)	0.455	0.92	−0.12	DOWN
C14:2-OH	0.01 (0.00)	0.01 (0.00)	0.125	0.87	−0.21	DOWN
C16	0.02 (0.00)	0.02 (0.01)	0.006	1.44	0.52	UP
C16:1	0.02 (0.00)	0.02 (0.00)	0.171	0.96	−0.07	DOWN
C16:1-OH	0.01 (0.00)	0.01 (0.00)	0.046	0.88	−0.18	DOWN
C16:2	0.01 (0.00)	0.01 (0.00)	0.901	1.03	0.04	UP
C16:2-OH	0.01 (0.00)	0.01 (0.00)	0.034	0.82	−0.29	DOWN
C16-OH	0.01 (0.00)	0.01 (0.00)	0.772	0.98	−0.03	DOWN
C18	0.03 (0.01)	0.04 (0.01)	0.740	1.02	0.03	UP
C18:1	0.01 (0.00)	0.02 (0.01)	0.361	1.15	0.20	UP
C18:1-OH	0.01 (0.00)	0.01 (0.00)	0.213	1.10	0.14	UP
C18:2	0.01 (0.00)	0.01 (0.00)	0.772	0.97	−0.04	DOWN
lyso PC a C14:0	1.43 (0.30)	1.16 (0.30)	0.038	0.81	−0.31	DOWN
lyso PC a C16:0	32.13 (6.27)	26.34 (9.28)	0.074	0.82	−0.29	DOWN
lyso PC a C16:1	1.89 (0.41)	1.46 (0.60)	0.046	0.77	−0.37	DOWN
lyso PC a C17:0	1.84 (0.48)	1.47 (0.63)	0.125	0.80	−0.32	DOWN
lyso PC a C18:0	22.09 (5.35)	20.06 (6.41)	0.507	0.91	−0.14	DOWN
lyso PC a C18:1	19.97 (4.15)	14.76 (6.14)	0.014	0.74	−0.44	DOWN
lyso PC a C18:2	45.24 (10.62)	31.89 (15.21)	0.014	0.70	−0.50	DOWN
lyso PC a C20:3	4.07 (1.07)	3.95 (1.45)	1.000	0.97	−0.04	DOWN
lyso PC a C20:4	2.66 (0.70)	2.02 (0.71)	0.034	0.76	−0.40	DOWN
lyso PC a C24:0	0.11 (0.03)	0.10 (0.03)	0.245	0.89	−0.17	DOWN
lyso PC a C26:0	0.20 (0.09)	0.15 (0.06)	0.263	0.74	−0.43	DOWN
lyso PC a C26:1	0.07 (0.02)	0.05 (0.02)	0.013	0.72	−0.46	DOWN
lyso PC a C28:0	0.41 (0.09)	0.28 (0.10)	0.001	0.68	−0.57	DOWN
lyso PC a C28:1	0.56 (0.12)	0.42 (0.19)	0.009	0.76	−0.40	DOWN
PC aa 32:2	15.55 (3.35)	14.41 (5.02)	0.384	0.93	−0.11	DOWN
PC aa 36:0	26.57 (4.35)	22.99 (7.49)	0.171	0.87	−0.21	DOWN
PC aa 36:6	4.09 (0.95)	4.38 (1.52)	0.740	1.07	0.10	UP
PC aa 38:0	4.08 (0.68)	3.33 (1.11)	0.042	0.82	−0.29	DOWN
PC aa 38:6	4.96 (0.76)	4.84 (1.53)	0.678	0.98	−0.04	DOWN
PC aa 40:1	0.50 (0.08)	0.47 (0.14)	0.772	0.93	−0.11	DOWN
PC aa 40:2	2.05 (0.35)	1.62 (0.59)	0.020	0.79	−0.34	DOWN
PC aa 40:6	2.83 (0.51)	3.01 (1.05)	0.934	1.06	0.09	UP
PC ae 36:0	4.29 (0.77)	3.76 (1.14)	0.171	0.88	−0.19	DOWN
PC ae 40:6	1.57 (0.25)	1.51 (0.43)	0.619	0.96	−0.06	DOWN
SM (OH) 14:1	14.95 (2.74)	12.29 (3.58)	0.042	0.82	−0.28	DOWN
SM 16:0	169.89 (28.24)	152.9 (45.5)	0.135	0.90	−0.15	DOWN
SM 16:1	18.93 (3.07)	16.25 (4.25)	0.042	0.86	−0.22	DOWN
SM (OH) 16:1	18.14 (3.01)	14.60 (4.70)	0.006	0.81	−0.31	DOWN
SM 18:0	28.43 (4.66)	23.32 (6.97)	0.009	0.82	−0.29	DOWN
SM 18:1	30.00 (5.63)	24.82 (10.86)	0.014	0.83	−0.27	DOWN
SM 20:2	3.62 (0.70)	3.25 (1.54)	0.147	0.90	−0.15	DOWN
SM (OH) 22:1	31.52 (6.47)	26.79 (8.24)	0.089	0.85	−0.23	DOWN
SM (OH) 22:2	15.11 (2.71)	13.14 (3.41)	0.062	0.87	−0.20	DOWN
SM (OH) 24:1	3.38 (0.55)	2.98 (0.89)	0.229	0.88	−0.18	DOWN
Glucose	3442.97 (281.65)	3909.23 (740.35)	0.062	1.14	0.18	UP
Alanine	285.20 (56.09)	256.20 (57.64)	0.106	0.90	−0.15	DOWN
Arginine	124.89 (25.88)	148.99 (32.75)	0.042	1.19	0.25	UP
Asparagine	36.41 (9.17)	32.33 (8.96)	0.213	0.89	−0.17	DOWN
Aspartic acid	7.86 (2.19)	11.48 (3.08)	0.002	1.46	0.55	UP
Citrulline	83.22 (22.63)	78.83 (11.98)	0.983	0.95	−0.08	DOWN
Glutamine	276.87 (43.57)	281.73 (42.49)	0.836	1.02	0.03	UP
Glutamic acid	64.47 (9.87)	69.47 (17.03)	0.361	1.08	0.11	UP
Glycine	389.07 (64.48)	328.6 (64.44)	0.010	0.84	−0.24	DOWN
Histidine	54.22 (10.86)	63.29 (13.31)	0.106	1.17	0.22	UP
Isoleucine	117.41 (27.96)	122.04 (26.49)	0.455	1.04	0.06	UP
Leucine	181.07 (45.40)	197.33 (59.44)	0.384	1.09	0.12	UP
Lysine	73.73 (20.73)	86.87 (27.54)	0.152	1.18	0.24	UP
Methionine	22.79 (5.85)	24.58 (5.06)	0.431	1.08	0.11	UP
Ornithine	47.94 (12.80)	55.15 (16.22)	0.221	1.15	0.20	UP
Phenylalanine	49.81 (10.19)	63.54 (17.60)	0.025	1.28	0.35	UP
Proline	113.59 (22.77)	101.56 (25.07)	0.097	0.89	−0.16	DOWN
Serine	90.77 (21.55)	79.79 (17.49)	0.130	0.88	−0.19	DOWN
Threonine	100.71 (27.61)	97.50 (28.56)	0.885	0.97	−0.05	DOWN
Tryptophan	49.03 (8.15)	51.68 (11.87)	0.520	1.05	0.08	UP
Tyrosine	70.37 (18.02)	68.43 (19.45)	0.709	0.97	−0.04	DOWN
Valine	205.47 (59.31)	239.73 (60.86)	0.110	1.17	0.22	UP
Acetyl-ornithine	4.01 (1.88)	3.58 (1.08)	0.967	0.89	−0.16	DOWN
alpha-Aminoadipic acid	1.95 (0.51)	2.11 (1.24)	0.868	1.08	0.11	UP
alpha-Ketoglutaric acid	22.82 (10.13)	22.97 (7.40)	0.590	1.01	0.01	UP
ADMA	0.65 (0.11)	0.75 (0.17)	0.085	1.14	0.19	UP
beta-Hydroxybutyric acid	909.13 (353.55)	721.00 (305.24)	0.135	0.79	−0.33	DOWN
Betaine	82.52 (38.26)	120.57 (67.06)	0.071	1.46	0.55	UP
Butyric acid	18.17 (8.80)	12.77 (8.58)	0.101	0.70	−0.51	DOWN
Carnosine	21.48 (8.82)	15.45 (5.30)	0.054	0.72	−0.48	DOWN
Choline	10.40 (3.21)	14.01 (3.30)	0.007	1.35	0.43	UP
Citric acid	282.40 (70.10)	227.92 (90.71)	0.089	0.81	−0.31	DOWN
Creatine	248.80 (28.53)	235.20 (39.61)	0.319	0.95	−0.08	DOWN
Creatinine	75.09 (11.60)	74.33 (11.64)	0.772	0.99	−0.01	DOWN
Fumaric acid	1.92 (1.02)	1.50 (0.93)	0.152	0.78	−0.36	DOWN
Hippuric acid	65.31 (13.66)	65.42 (18.20)	0.934	1.00	0.00	---
Indole acetic acid	0.59 (0.25)	0.76 (0.50)	0.481	1.29	0.36	UP
Isobutyric acid	4.82 (1.71)	6.91 (4.21)	0.147	1.43	0.52	UP
Kynurenine	9.06 (2.72)	8.60 (4.12)	0.648	0.95	−0.07	DOWN
Lactic acid	1489.93 (729.91)	1514.73 (720.62)	0.917	1.02	0.02	UP
Methionine-sulfoxide	2.47 (0.62)	2.14 (0.71)	0.237	0.87	−0.21	DOWN
Methylhistidine	9.37 (2.63)	10.02 (2.49)	0.468	1.07	0.10	UP
Methylmalonic acid	0.60 (0.13)	0.64 (0.47)	0.263	1.07	0.09	UP
Propionic acid	35.89 (18.95)	29.61 (15.44)	0.431	0.83	−0.28	DOWN
Pyruvic acid	57.17 (13.86)	67.69 (16.98)	0.074	1.18	0.24	UP
Serotonin	7.25 (5.04)	8.08 (6.42)	0.934	1.12	0.16	UP
Spermidine	0.29 (0.12)	0.29 (0.14)	0.836	1.02	0.02	UP
Succinic acid	1.48 (0.31)	1.37 (0.43)	0.130	0.93	−0.11	DOWN
Taurine	75.57 (20.50)	73.01 (14.75)	0.803	0.97	−0.05	DOWN
TDMA	1.92 (0.41)	1.79 (0.36)	0.836	0.93	−0.10	DOWN
trans-Hydroxyproline	12.57 (2.65)	10.83 (2.49)	0.018	0.86	−0.22	DOWN
Trimethylamine N-oxide	47.05 (33.10)	46.28 (44.15)	0.619	0.98	−0.02	DOWN
Uric acid	37.16 (12.94)	46.48 (24.56)	0.158	1.25	0.32	UP

^1^ C2: Acetyl-L-carnitine; C3: Propionyl-L-carnitine; C4: Butyryl-L-carnitine; C5: Valeryl-L-carnitine; C10: Decanoyl-L-carnitine; C16: Hexadecanoyl-L-carnitine; C18: Octadecanoyl-L-carnitine; lysoPC a: lysophosphatidylcholine acyl; PC aa: phosphatidylcholine diacyl; PC ae: phosphatidylcholine acyl-alkyl; lysoPC a, PC aa, and PC ae are glycerophospholipids; ADMA: asymmetric dimethylarginine; TDMA: total dimethylarginine. ^2^ Log_2_(FC): log_2_ fold change.

**Table 2:** Concentrations of serum metabolites [Mean (SD)] in clinically healthy (CON) and leukemia (LEU) cows −4 weeks prepartum as determined by DI/LC-MS/MS.

−4 Weeks Prepartum						
Metabolite, μM ^1^	CON (SD)	LEU (SD)	*p*-Value	FC	Log_2_(FC) ^2^	LEU/CON
C0	4.76 (1.62)	4.18 (1.20)	0.431	0.88	−0.19	DOWN
C2	2.01 (0.57)	1.93 (0.46)	0.709	0.96	−0.06	DOWN
C3	0.19 (0.05)	0.18 (0.04)	0.590	0.92	−0.11	DOWN
C3:1	0.02 (0.00)	0.02 (0.01)	0.340	0.93	−0.11	DOWN
C3-OH	0.02 (0.01)	0.02 (0.00)	0.002	0.69	−0.53	DOWN
C4	0.10 (0.02)	0.10 (0.03)	0.648	1.04	0.06	UP
C4:1	0.02 (0.00)	0.01 (0.00)	0.678	0.96	−0.06	DOWN
C4-OH (C3-DC)	0.03 (0.01)	0.03 (0.01)	0.561	0.98	−0.03	DOWN
C5	0.08 (0.01)	0.07 (0.02)	0.184	0.89	−0.17	DOWN
C5:1	0.01 (0.00)	0.01 (0.00)	0.020	0.81	−0.31	DOWN
C5:1-DC (C6-OH)	0.01 (0.00)	0.01 (0.00)	0.147	1.13	0.17	UP
C5-DC	0.01 (0.00)	0.01 (0.00)	0.407	0.94	−0.09	DOWN
C5-M-DC	0.02 (0.00)	0.02 (0.00)	0.005	1.15	0.20	UP
C5-OH (C3-DC-M)	0.06 (0.03)	0.08 (0.02)	0.038	1.42	0.51	UP
C6	0.03 (0.01)	0.05 (0.04)	0.081	1.58	0.66	UP
C6:1	0.02 (0.01)	0.03 (0.01)	0.619	1.12	0.16	UP
C8	0.01 (0.00)	0.01 (0.01)	0.590	1.08	0.11	UP
C9	0.01 (0.00)	0.01 (0.00)	0.147	0.92	−0.12	DOWN
C10	0.04 (0.01)	0.03 (0.01)	0.534	0.9	−0.15	DOWN
C10:1	0.11 (0.06)	0.08 (0.03)	0.300	0.68	−0.56	DOWN
C10:2	0.02 (0.01)	0.02 (0.00)	0.901	0.95	−0.08	DOWN
C12	0.02 (0.00)	0.03 (0.01)	0.590	1.14	0.19	UP
C12:1	0.05 (0.03)	0.04 (0.01)	0.281	0.73	−0.46	DOWN
C12-DC	0.01 (0.00)	0.01 (0.00)	0.281	1.09	0.13	UP
C14	0.01 (0.00)	0.01 (0.01)	0.407	1.08	0.11	UP
C14:1	0.05 (0.02)	0.04 (0.01)	0.068	0.83	−0.26	DOWN
C14:1-OH	0.01 (0.00)	0.01 (0.00)	0.431	1.08	0.11	UP
C14:2	0.01 (0.00)	0.01 (0.00)	0.648	1.07	0.10	UP
C14:2-OH	0.01 (0.00)	0.01 (0.00)	1.000	0.96	−0.06	DOWN
C16	0.02 (0.00)	0.03 (0.01)	0.709	1.15	0.20	UP
C16:1	0.02 (0.00)	0.02 (0.00)	0.213	0.97	−0.04	DOWN
C16:1-OH	0.01 (0.00)	0.01 (0.00)	0.868	0.98	−0.03	DOWN
C16:2	0.01 (0.00)	0.01 (0.00)	0.507	0.96	−0.06	DOWN
C16:2-OH	0.01 (0.00)	0.01 (0.00)	0.135	0.87	−0.21	DOWN
C16-OH	0.01 (0.00)	0.01 (0.00)	0.056	0.85	−0.23	DOWN
C18	0.03 (0.01)	0.03 (0.01)	0.967	1.08	0.11	UP
C18:1	0.02 (0.00)	0.02 (0.01)	0.320	1.23	0.30	UP
C18:1-OH	0.01 (0.00)	0.01 (0.00)	0.184	0.86	−0.22	DOWN
C18:2	0.01 (0.00)	0.01 (0.00)	0.934	0.98	−0.03	DOWN
lyso PC a C14:0	0.84 (0.25)	0.76 (0.21)	0.320	0.91	−0.14	DOWN
lyso PC a C16:0	17.78 (4.36)	17.35 (4.67)	0.868	0.98	−0.03	DOWN
lyso PC a C16:1	1.13 (0.37)	1.16 (0.42)	0.481	1.03	0.04	UP
lyso PC a C17:0	1.29 (0.27)	1.29 (0.48)	0.868	1.00	0.01	UP
lyso PC a C18:0	15.89 (3.24)	13.33 (2.88)	0.042	0.84	−0.25	DOWN
lyso PC a C18:1	11.18 (3.40)	10.75 (3.63)	0.967	0.96	−0.06	DOWN
lyso PC a C18:2	19.96 (5.95)	18.42 (6.67)	0.534	0.92	−0.12	DOWN
lyso PC a C20:3	3.32 (1.02)	2.79 (0.81)	0.135	0.84	−0.25	DOWN
lyso PC a C20:4	1.82 (0.54)	1.77 (0.65)	0.868	0.97	−0.04	DOWN
lyso PC a C24:0	0.11 (0.03)	0.10 (0.01)	0.507	0.93	−0.10	DOWN
lyso PC a C26:0	0.08 (0.03)	0.07 (0.02)	0.229	0.83	−0.27	DOWN
lyso PC a C26:1	0.04 (0.01)	0.04 (0.01)	0.803	0.98	−0.03	DOWN
lyso PC a C28:0	0.23 (0.07)	0.21 (0.08)	0.245	0.90	−0.15	DOWN
lyso PC a C28:1	0.31 (0.11)	0.27 (0.13)	0.171	0.86	−0.22	DOWN
PC aa 32:2	11.85 (2.90)	9.83 (3.24)	0.074	0.83	−0.27	DOWN
PC aa 36:0	14.48 (2.02)	11.94 (4.01)	0.028	0.82	−0.28	DOWN
PC aa 36:6	3.59 (0.93)	2.98 (1.02)	0.074	0.83	−0.27	DOWN
PC aa 38:0	1.93 (0.30)	1.39 (0.58)	0.005	0.72	−0.47	DOWN
PC aa 38:6	3.45 (0.68)	2.75 (0.63)	0.010	0.80	−0.32	DOWN
PC aa 40:1	0.40 (0.08)	0.33 (0.09)	0.046	0.83	−0.26	DOWN
PC aa 40:2	0.99 (0.18)	0.71 (0.25)	0.004	0.72	−0.48	DOWN
PC aa 40:6	2.54 (0.43)	2.21 (0.57)	0.081	0.87	−0.20	DOWN
PC ae 36:0	3.38 (0.68)	2.71 (0.91)	0.034	0.8	−0.32	DOWN
PC ae 40:6	1.15 (0.18)	0.95 (0.23)	0.011	0.83	−0.28	DOWN
SM (OH) 14:1	9.30 (2.06)	7.78 (2.10)	0.068	0.84	−0.26	DOWN
SM 16:0	101.36 (25.24)	89.70 (21.76)	0.171	0.88	−0.18	DOWN
SM 16:1	11.21 (2.63)	9.70 (2.44)	0.135	0.87	−0.21	DOWN
SM (OH) 16:1	9.92 (2.45)	8.86 (2.55)	0.300	0.89	−0.16	DOWN
SM 18:0	17.23 (4.27)	15.98 (3.88)	0.340	0.93	−0.11	DOWN
SM 18:1	16.60 (5.51)	15.45 (4.87)	0.740	0.93	−0.10	DOWN
SM 20:2	2.41 (0.95)	2.18 (0.79)	0.678	0.9	−0.15	DOWN
SM (OH) 22:1	18.13 (4.72)	17.42 (4.74)	0.648	0.96	−0.06	DOWN
SM (OH) 22:2	10.01 (2.53)	9.33 (2.50)	0.361	0.93	−0.10	DOWN
SM (OH) 24:1	2.27 (0.63)	2.32 (0.56)	0.772	1.02	0.03	UP
Glucose	3900.8 (423.3)	3855.92 (376.37)	0.678	0.99	−0.02	DOWN
Alanine	2.16 (1.32)	1.99 (0.67)	0.868	0.92	−0.12	DOWN
Arginine	30.46 (4.57)	29.38 (7.37)	0.590	0.96	−0.05	DOWN
Asparagine	8.15 (2.88)	9.89 (2.68)	0.130	1.21	0.28	UP
Aspartic acid	1.07 (0.19)	1.02 (0.13)	0.220	0.95	−0.07	DOWN
Citrulline	85.98 (16.86)	85.55 (14.21)	0.836	0.99	−0.01	DOWN
Glutamine	330.33 (43.70)	317.20 (46.41)	0.350	0.96	−0.06	DOWN
Glutamic acid	68.43 (21.47)	69.57 (19.99)	0.885	1.02	0.02	UP
Glycine	262.47 (31.35)	236.60 (26.17)	0.026	0.90	−0.15	DOWN
Histidine	71.49 (9.49)	68.01 (10.49)	0.395	0.95	−0.07	DOWN
Isoleucine	145.27 (17.73)	149.87 (24.80)	0.618	1.03	0.04	UP
Leucine	232.53 (67.47)	244.47 (46.21)	0.213	1.05	0.07	UP
Lysine	96.77 (30.14)	103.05 (21.40)	0.290	1.06	0.09	UP
Methionine	29.46 (5.08)	27.95 (4.84)	0.520	0.95	−0.08	DOWN
Ornithine	63.85 (11.38)	60.23 (10.44)	0.395	0.94	−0.08	DOWN
Phenylalanine	63.49 (9.68)	66.11 (7.48)	0.178	1.04	0.06	UP
Proline	97.81 (14.53)	81.47 (16.86)	0.012	0.83	−0.26	DOWN
Serine	74.99 (14.60)	70.39 (13.21)	0.633	0.94	−0.09	DOWN
Threonine	2.22 (0.33)	2.47 (0.40)	0.054	1.11	0.16	UP
Tryptophan	43.63 (7.99)	41.67 (6.73)	0.967	0.96	−0.07	DOWN
Tyrosine	64.95 (15.17)	64.10 (12.77)	0.934	0.99	−0.02	DOWN
Valine	294.07 (48.43)	262.80 (52.10)	0.184	0.89	−0.16	DOWN
Acetyl-ornithine	3.86 (1.22)	3.28 (1.59)	0.272	0.85	−0.24	DOWN
alpha-Aminoadipic acid	16.94 (5.38)	19.44 (3.49)	0.068	1.15	0.20	UP
alpha-Ketoglutaric acid	148.47 (25.11)	136.33 (26.66)	0.089	0.92	−0.12	DOWN
ADMA	240.07 (32.79)	212.73 (41.04)	0.026	0.89	−0.17	DOWN
beta-Hydroxybutyric acid	676.20 (261.66)	520.80 (147.68)	0.078	0.77	−0.38	DOWN
Betaine	164.53 (37.37)	150.69 (46.30)	0.494	0.92	−0.13	DOWN
Butyric acid	7.26 (2.43)	5.35 (2.73)	0.065	0.74	−0.44	DOWN
Carnosine	13.01 (4.63)	9.26 (4.83)	0.033	0.71	−0.49	DOWN
Choline	11.75 (2.89)	12.53 (5.37)	0.724	1.07	0.09	UP
Citric acid	292.67 (110.61)	227.25 (82.03)	0.068	0.78	−0.36	DOWN
Creatine	242.67 (33.07)	240.07 (33.93)	0.884	0.99	−0.02	DOWN
Creatinine	75.09 (11.60)	74.33 (11.64)	0.772	0.99	−0.01	DOWN
Fumaric acid	1.07 (0.24)	1.15 (0.22)	0.372	1.07	0.10	UP
Hippuric acid	71.57 (15.24)	66.83 (15.73)	0.351	0.93	−0.10	DOWN
Indole acetic acid	0.40 (0.16)	0.36 (0.18)	0.35	0.92	−0.13	DOWN
Isobutyric acid	5.22 (1.62)	4.65 (1.48)	0.351	0.89	−0.17	DOWN
Kynurenine	6.66 (2.40)	7.15 (3.78)	0.633	1.07	0.10	UP
Lactic acid	1420.53 (680.05)	1362.27 (819.95)	0.361	0.96	−0.06	DOWN
Methionine-sulfoxide	2.82 (0.81)	1.79 (0.58)	0.001	0.64	−0.65	DOWN
Methylhistidine	13.87 (4.22)	12.91 (3.34)	0.756	0.93	−0.10	DOWN
Methylmalonic acid	0.35 (0.13)	0.37 (0.18)	0.836	1.07	0.09	UP
Propionic acid	19.64 (7.54)	16.83 (7.92)	0.418	0.86	−0.22	DOWN
Pyruvic acid	67.15 (17.04)	69.96 (23.32)	0.917	1.04	0.06	UP
Serotonin	7.94 (9.72)	4.88 (6.11)	0.290	0.61	−0.70	DOWN
Spermidine	0.31 (0.17)	0.22 (0.10)	0.165	0.70	−0.51	DOWN
Succinic acid	1.15 (0.27)	1.07 (0.24)	0.547	0.93	−0.11	DOWN
Taurine	71.39 (23.64)	67.73 (21.07)	0.756	0.95	−0.08	DOWN
TDMA	98.41 (24.02)	89.70 (16.86)	0.885	0.91	−0.13	DOWN
trans-Hydroxyproline	15.41 (2.86)	13.13 (3.25)	0.085	0.85	−0.23	DOWN
Trimethylamine N-oxide	23.65 (24.39)	13.34 (10.02)	0.229	0.56	−0.83	DOWN
Uric acid	37.38 (28.74)	40.21 (27.23)	0.372	1.08	0.11	UP

^1^ C2: Acetyl-L-carnitine; C3: Propionyl-L-carnitine; C4: Butyryl-L-carnitine; C5: Valeryl-L-carnitine; C10: Decanoyl-L-carnitine; C16: Hexadecanoyl-L-carnitine; C18: Octadecanoyl-L-carnitine; lysoPC a: lysophosphatidylcholine acyl; PC aa: phosphatidylcholine diacyl; PC ae: phosphatidylcholine acyl-alkyl; lysoPC a, PC aa, and PC ae are glycerophospholipids; ADMA: asymmetric dimethylarginine; TDMA: total dimethylarginine. ^2^ Log_2_(FC): log_2_ fold change.

**Table 3:** Concentrations of urine metabolites [Mean (SD)] in clinically healthy (CON) and leukemia (LEU) cows −8 weeks prepartum as determined by DI/LC-MS/MS.

−8 Weeks Prepartum						
Metabolite, μM ^1^	CON (SD)	LEU (SD)	*p*-Value	FC	Log_2_(FC) ^2^	LEU/CON
C0	1.12 (0.61)	1.26 (0.55)	0.561	1.12	0.17	UP
C2	0.34 (0.10)	0.45 (0.15)	0.081	1.33	0.41	UP
C3	0.03 (0.01)	0.03 (0.02)	0.534	1.11	0.15	UP
C3:1	0.02 (0.01)	0.02 (0.01)	0.407	1.17	0.23	UP
C3-OH	0.07 (0.02)	0.07 (0.02)	0.431	1.05	0.07	UP
C4	0.19 (0.10)	0.28 (0.14)	0.023	1.49	0.58	UP
C4:1	0.06 (0.02)	0.07 (0.03)	0.281	1.16	0.22	UP
C4-OH (C3-DC)	0.07 (0.02)	0.07 (0.02)	0.648	0.95	−0.08	DOWN
C5	0.12 (0.05)	0.11 (0.05)	0.836	0.93	−0.10	DOWN
C5:1	0.14 (0.06)	0.14 (0.05)	0.868	1.03	0.04	UP
C5:1-DC (C6-OH)	0.03 (0.01)	0.04 (0.01)	1.000	1.04	0.06	UP
C5-DC	0.03 (0.01)	0.03 (0.01)	0.803	1.05	0.07	UP
C5-M-DC	0.04 (0.01)	0.05 (0.01)	0.074	1.21	0.28	UP
C5-OH (C3-DC-M)	0.08 (0.03)	0.09 (0.02)	0.740	1.03	0.04	UP
C6	0.08 (0.04)	0.08 (0.03)	0.590	1.06	0.09	UP
C6:1	0.07 (0.03)	0.08 (0.02)	0.455	1.11	0.14	UP
C7-DC	0.03 (0.02)	0.03 (0.01)	0.934	0.93	−0.11	DOWN
C8	0.03 (0.02)	0.04 (0.02)	0.056	1.34	0.43	UP
C9	0.10 (0.06)	0.10 (0.04)	0.740	0.99	−0.02	DOWN
C10	0.09 (0.02)	0.12 (0.03)	0.004	1.31	0.39	UP
C10:1	0.12 (0.04)	0.17 (0.04)	0.010	1.37	0.45	UP
C10:2	0.04 (0.01)	0.05 (0.01)	0.051	1.20	0.27	UP
C12	0.07 (0.02)	0.12 (0.10)	0.003	1.88	0.91	UP
C12:1	0.11 (0.06)	0.17 (0.07)	0.018	1.47	0.55	UP
Glucose	1244.85 (634.77)	2479.37 (1200.03)	0.003	1.99	0.99	UP
Alanine	248.59 (118.99)	197.04 (97.34)	0.340	0.79	−0.34	DOWN
Arginine	9.08 (5.12)	12.74 (8.54)	0.213	1.40	0.49	UP
Asparagine	8.76 (4.69)	11.63 (7.57)	0.281	1.33	0.41	UP
Aspartic acid	132.49 (103.51)	131.56 (72.13)	0.709	0.99	−0.01	DOWN
Citrulline	4.55 (3.59)	7.07 (4.38)	0.046	1.55	0.63	UP
Glutamine	258.05 (165.24)	292.15 (177.73)	0.678	1.13	0.18	UP
Glutamic acid	103.23 (66.24)	92.03 (48.27)	0.709	0.89	−0.17	DOWN
Glycine	351.55 (437.92)	250.31 (221.53)	1.000	0.71	−0.49	DOWN
Histidine	77.01 (60.57)	82.78 (52.01)	0.561	1.07	0.10	UP
Isoleucine	12.00 (12.97)	10.78 (7.31)	0.534	0.90	−0.16	DOWN
Leucine	6.79 (3.74)	8.65 (4.06)	0.120	1.27	0.35	UP
Lysine	53.07 (34.93)	60.67 (33.51)	0.455	1.14	0.19	UP
Methionine	2.71 (0.68)	3.13 (0.91)	0.191	1.16	0.21	UP
Ornithine	17.11 (10.12)	19.45 (6.95)	0.237	1.14	0.19	UP
Phenylalanine	11.30 (6.90)	14.64 (11.75)	0.407	1.30	0.37	UP
Proline	7.41 (4.52)	5.67 (2.77)	0.520	0.76	−0.39	DOWN
Serine	74.59 (46.99)	84.83 (40.33)	0.320	1.14	0.19	UP
Threonine	67.11 (39.41)	68.59 (35.24)	0.917	1.02	0.03	UP
Tryptophan	27.26 (16.29)	25.17 (12.73)	0.885	0.92	−0.12	DOWN
Tyrosine	21.37 (10.49)	22.40 (9.46)	0.678	1.05	0.07	UP
Valine	10.50 (6.91)	11.54 (4.60)	0.320	1.10	0.14	UP
Acetyl-ornithine	51.89 (34.54)	56.36 (25.04)	0.340	1.09	0.12	UP
ADMA	3.07 (1.87)	6.03 (4.57)	0.023	1.97	0.98	UP
alpha-Aminoadipic acid	107.94 (70.37)	98.35 (56.85)	0.772	0.91	−0.13	DOWN
alpha-Ketoglutaric acid	47.65 (124.98)	53.00 (160.42)	0.619	1.11	0.15	UP
beta-Hydroxybutyric acid	664.87 (1242.58)	286.33 (525.75)	0.213	0.43	−1.22	DOWN
Betaine	113.17 (89.01)	204.88 (164.69)	0.097	1.81	0.86	UP
Butyric acid	43.40 (39.29)	34.30 (38.63)	0.213	0.79	−0.34	DOWN
Carnosine	16.37 (11.21)	14.72 (8.95)	0.561	0.90	−0.15	DOWN
Choline	26.75 (25.65)	50.75 (63.00)	0.351	1.90	0.92	UP
Citric acid	976.90 (1968.16)	984.5 (1735.92)	0.619	1.01	0.01	UP
Creatine	3140.67 (1603.8)	4036.13 (1691.91)	0.184	1.29	0.36	UP
Creatinine	8130.67 (4491.35)	9936.67 (5070.74)	0.300	1.22	0.29	UP
Hippuric acid	13,707.33 (4854.71)	12,136.00 (2795.60)	0.480	0.89	−0.18	DOWN
Histamine	0.10 (0.06)	0.11 (0.06)	0.633	1.07	0.10	UP
Homovanillic acid	6.59 (4.19)	7.93 (5.62)	0.431	1.20	0.27	UP
Indole acetic acid	66.79 (54.74)	66.61 (45.70)	0.852	1.00	0.00	---
Isobutyric acid	6.01 (4.30)	10.88 (15.96)	0.619	1.81	0.86	UP
Kynurenine	1.37 (1.11)	1.06 (0.63)	0.709	0.77	−0.37	DOWN
Lactic acid	108.56 (146.26)	96.51 (73.37)	0.431	0.89	−0.17	DOWN
Methionine-sulfoxide	2.65 (2.19)	2.29 (1.53)	0.967	0.86	−0.21	DOWN
Methylhistidine	224.15 (148.69)	266.00 (171.06)	0.431	1.19	0.25	UP
Methylmalonic acid	23.00 (17.27)	24.75 (23.39)	0.885	1.08	0.11	UP
p-Hydroxyhippuric acid	43.62 (39.85)	39.65 (28.06)	0.950	0.91	−0.14	DOWN
Putrescine	0.42 (0.22)	0.62 (0.57)	0.852	1.47	0.55	UP
Pyruvic acid	5.01 (4.48)	6.21 (4.36)	0.141	1.24	0.31	UP
Sarcosine	2.72 (2.26)	4.14 (3.41)	0.213	1.52	0.61	UP
Serotonin	1.43 (0.84)	1.55 (0.74)	0.419	1.09	0.12	UP
Spermidine	0.08 (0.05)	0.25 (0.57)	0.237	3.09	1.63	UP
Spermine	0.08 (0.03)	0.10 (0.05)	0.135	1.36	0.45	UP
Succinic acid	22.30 (23.99)	26.10 (20.52)	0.330	1.17	0.23	UP
Taurine	492.27 (512.53)	653.27 (522.13)	0.263	1.33	0.41	UP
TDMA	20.38 (10.71)	26.82 (14.60)	0.229	1.32	0.40	UP
trans-Hydroxyproline	1.61 (1.53)	1.35 (0.79)	0.852	0.84	−0.26	DOWN
Trimethylamine N-oxide	3960.93 (3391.24)	4052.87 (4086.49)	1.000	1.02	0.03	UP
Tyramine	0.08 (0.08)	0.14 (0.12)	0.088	1.75	0.81	UP
Uric acid	4122.67 (2408.00)	4300.00 (2455.60)	0.772	1.04	0.06	UP

^1^ C2: Acetyl-L-carnitine; C3: Propionyl-L-carnitine; C4: Butyryl-L-carnitine; C5: Valeryl-L-carnitine; C10: Decanoyl-L-carnitine; C16: Hexadecanoyl-L-carnitine; C18: Octadecanoyl-L-carnitine; ADMA: asymmetric dimethylarginine; TDMA: total dimethylarginine. ^2^ Log_2_(FC): log_2_ fold change.

**Table 4:** Concentrations of urine metabolites [Mean (SD)] in clinically healthy (CON) and leukemia (LEU) cows −4 weeks prepartum as determined by DI/LC-MS/MS.

−4 Weeks Prepartum						
Metabolite, μM ^1^	CON (SD)	LEU (SD)	*p*-Value	FC	Log_2_(FC) ^2^	LEU/CON
C0	1.17 (0.32)	1.27 (0.49)	0.561	1.09	0.12	UP
C2	0.64 (0.32)	0.52 (0.21)	0.431	0.82	−0.29	DOWN
C3	0.06 (0.01)	0.05 (0.01)	0.056	0.91	−0.14	DOWN
C3:1	0.05 (0.01)	0.05 (0.02)	0.648	1.00	−0.01	DOWN
C3-OH	0.07 (0.02)	0.07 (0.02)	1.000	1.01	0.02	UP
C4	0.38 (0.22)	0.42 (0.19)	0.534	1.11	0.14	UP
C4:1	0.07 (0.02)	0.07 (0.02)	0.619	0.99	−0.02	DOWN
C4-OH (C3-DC)	0.07 (0.02)	0.07 (0.02)	0.901	1.00	0.00	---
C5	0.15 (0.06)	0.12 (0.04)	0.481	0.84	−0.25	DOWN
C5:1	0.17 (0.03)	0.20 (0.13)	0.709	1.20	0.26	UP
C5:1-DC (C6-OH)	0.04 (0.01)	0.03 (0.01)	0.534	0.97	−0.05	DOWN
C5-DC	0.03 (0.01)	0.03 (0.02)	0.709	1.13	0.17	UP
C5-M-DC	0.05 (0.01)	0.05 (0.02)	0.934	1.02	0.02	UP
C5-OH (C3-DC-M)	0.11 (0.02)	0.12 (0.04)	0.934	1.06	0.08	UP
C6	0.09 (0.02)	0.09 (0.03)	0.534	0.92	−0.11	DOWN
C6:1	0.08 (0.02)	0.08 (0.02)	0.384	1.04	0.05	UP
C7-DC	0.03 (0.01)	0.04 (0.02)	0.245	1.25	0.32	UP
C8	0.04 (0.01)	0.04 (0.01)	0.740	0.98	−0.03	DOWN
C9	0.12 (0.04)	0.13 (0.06)	0.740	1.08	0.12	UP
C10	0.13 (0.04)	0.13 (0.05)	0.901	0.98	−0.02	DOWN
C10:1	0.18 (0.04)	0.20 (0.05)	0.229	1.13	0.17	UP
C10:2	0.05 (0.02)	0.05 (0.03)	0.648	1.06	0.08	UP
C12	0.11 (0.05)	0.09 (0.06)	0.320	0.89	−0.17	DOWN
C12:1	0.21 (0.1)	0.20 (0.12)	0.648	0.93	−0.10	DOWN
Glucose	1600.88 (1218.21)	1253.64 (944.4)	0.431	0.78	−0.35	DOWN
Alanine	83.23 (25.77)	89.55 (38.33)	0.494	1.08	0.11	UP
Arginine	11.32 (3.30)	12.08 (5.55)	0.663	1.07	0.09	UP
Asparagine	9.52 (2.98)	10.86 (5.54)	0.395	1.14	0.19	UP
Aspartic acid	140.83 (60.83)	144.25 (73.88)	0.82	1.02	0.03	UP
Citrulline	6.76 (4.16)	8.41 (6.88)	0.709	1.24	0.32	UP
Glutamine	215.95 (85.79)	245.27 (136.20)	0.633	1.14	0.18	UP
Glutamic acid	57.76 (16.75)	62.01 (35.25)	0.885	1.07	0.10	UP
Glycine	73.81 (33.98)	83.26 (48.58)	0.431	1.13	0.17	UP
Histidine	64.61 (16.23)	75.67 (42.70)	0.431	1.17	0.23	UP
Isoleucine	6.07 (1.22)	6.82 (3.08)	0.330	1.12	0.17	UP
Leucine	9.15 (2.51)	10.43 (4.79)	0.633	1.14	0.19	UP
Lysine	50.96 (11.56)	58.83 (32.78)	0.604	1.15	0.21	UP
Methionine	3.23 (0.50)	3.29 (0.86)	0.836	1.02	0.03	UP
Ornithine	16.28 (5.10)	17.59 (9.46)	0.756	1.08	0.11	UP
Phenylalanine	11.04 (2.70)	13.21 (7.76)	0.372	1.20	0.26	UP
Proline	3.99 (1.21)	3.87 (1.79)	0.772	0.97	−0.04	DOWN
Serine	67.39 (23.68)	78.19 (33.86)	0.384	1.16	0.21	UP
Threonine	51.43 (17.40)	58.97 (37.45)	0.663	1.15	0.20	UP
Tryptophan	19.51 (6.70)	21.41 (14.62)	0.756	1.10	0.13	UP
Tyrosine	21.25 (5.43)	23.76 (13.32)	0.803	1.12	0.16	UP
Valine	10.45 (2.59)	11.64 (4.64)	0.395	1.11	0.16	UP
Acetyl-ornithine	46.31 (14.14)	62.25 (33.27)	0.135	1.34	0.43	UP
ADMA	6.78 (2.53)	7.73 (4.06)	0.507	1.14	0.19	UP
alpha-Aminoadipic acid	78.13 (32.65)	75.09 (28.38)	0.983	0.96	−0.06	DOWN
alpha-Ketoglutaric acid	9.66 (12.05)	52.06 (147.06)	0.184	5.39	2.43	UP
beta-Hydroxybutyric acid	133.04 (185.33)	83.27 (75.78)	0.934	0.63	−0.68	DOWN
Betaine	358.29 (286.02)	280.29 (235.62)	0.431	0.78	−0.35	DOWN
Butyric acid	7.68 (4.44)	9.1 (6.04)	0.494	1.19	0.25	UP
Carnosine	11 (3.75)	10.82 (5.46)	0.561	0.98	−0.02	DOWN
Choline	57.42 (39.46)	74.23 (109.13)	0.633	1.29	0.37	UP
Citric acid	321.2 (230.27)	609.29 (1345.26)	0.678	1.90	0.92	UP
Creatine	5900.8 (3108.32)	5826.27 (3548.61)	0.983	0.99	−0.02	DOWN
Creatinine	1440.00 (0.00)	1440.00 (0.00)	1.000	1.00	0.00	---
Hippuric acid	17,384.67 (5496.79)	19,588.00 (5948.72)	0.245	1.13	0.17	UP
Histamine	0.07 (0.04)	0.07 (0.06)	0.868	1.10	0.13	UP
Homovanillic acid	8.93 (4.04)	9.72 (5.95)	0.934	1.09	0.12	UP
Indole acetic acid	33.97 (17.46)	36.08 (12.41)	0.361	1.06	0.09	UP
Isobutyric acid	3.03 (1.40)	7.91 (8.13)	0.320	2.61	1.38	UP
Kynurenine	0.74 (0.18)	0.76 (0.41)	0.772	1.03	0.04	UP
Lactic acid	85.64 (34.87)	119.79 (141.47)	0.803	1.40	0.48	UP
Methionine-sulfoxide	3.15 (1.18)	3.15 (1.91)	0.619	1.00	0.00	---
Methylhistidine	249.20 (60.61)	292.11 (137.63)	0.263	1.17	0.23	UP
Methylmalonic acid	10.78 (5.42)	16.54 (9.67)	0.062	1.53	0.62	UP
p-Hydroxyhippuric acid	46.69 (33.28)	47.43 (53.63)	0.619	1.02	0.02	UP
Putrescine	1.14 (1.62)	0.82 (0.92)	0.455	0.71	−0.49	DOWN
Pyruvic acid	6.27 (2.64)	11.81 (12.14)	0.020	1.89	0.91	UP
Sarcosine	7.74 (5.35)	5.90 (4.68)	0.281	0.76	−0.39	DOWN
Serotonin	1.40 (0.33)	1.53 (0.75)	1.000	1.09	0.13	UP
Spermidine	0.11 (0.07)	0.13 (0.07)	0.431	1.17	0.23	UP
Spermine	0.12 (0.05)	0.11 (0.05)	0.917	0.97	−0.04	DOWN
Succinic acid	24.74 (21.09)	23.28 (25.5)	0.481	0.94	−0.09	DOWN
Taurine	425.51 (359.00)	370.12 (180.44)	0.803	0.87	−0.20	DOWN
TDMA	27.73 (7.04)	31.63 (15.98)	0.803	1.14	0.19	UP
trans-Hydroxyproline	2.03 (0.90)	2.13 (1.67)	0.648	1.05	0.07	UP
Trimethylamine N-oxide	1837.73 (1663.15)	2385.80 (1881.73)	0.407	1.30	0.38	UP
Tyramine	0.10 (0.06)	0.11 (0.07)	0.633	1.11	0.15	UP
Uric acid	3133.33 (1041.73)	4234.00 (2610.31)	0.351	1.35	0.43	UP

^1^ C2: Acetyl-L-carnitine; C3: Propionyl-L-carnitine; C4: Butyryl-L-carnitine; C5: Valeryl-L-carnitine; C10: Decanoyl-L-carnitine; C16: Hexadecanoyl-L-carnitine; C18: Octadecanoyl-L-carnitine; ADMA: asymmetric dimethylarginine; TDMA: total dimethylarginine. ^2^ Log_2_(FC): log_2_ fold change.

## Data Availability

The dataset generated and analyzed during the current study is not publicly accessible due to confidentiality agreements and ongoing intellectual property development. Researchers with specific inquiries about the data may contact the corresponding author to discuss the possibility of accessing anonymized or aggregated data subsets, subject to approval and appropriate non-disclosure agreements.

## Supplementary Materials

The following supporting information can be downloaded at: https://www.mdpi.com/article/10.3390/metabo14110624/s1, Table S1: Composition of the prepartum diet for dry-off cows; Table S2: Nutritional components in early lactation cow feeding.
