# Supplementary material for: Metabolic Fingerprinting of Blood and Urine of Dairy Cows Affected by Bovine Leukemia Virus: A Mass Spectrometry Approach
Source: Metabolites. 2024 Nov 14;14(11):624. doi: 10.3390/metabo14110624 (PMC11596772; doi:10.3390/metabo14110624)
Supplement: Supplementary file 1 [file metabolites-14-00624-s001.zip › metabolites-3300089-supplementary.pdf]

Supplementary Table S1. Composition of the Prepartum Diet for Dry-Off Cows.

Ingredient	Weight/Cow (kg)	DM 1 (%)	Final DMI 1 (kg)
Hay	5.50	85.14%	4.68
Oats	5.75	36.20%	2.08
Corn	8.84	30.30%	2.68
Protein	2.00	93.00%	1.86
Ground Barley	0.75	97.26%	0.66
Minerals	0.42	97.26%	0.41
Total	23.36	53.17%	12.37

[1]Dry matter intake (DMI) is computed by multiplying the percentage of dry matter (DM%) in the given feed by the weight (kg) of the offered feed. The daily DMI goal is established at 2% of the cow's body weight.

Supplementary Table S2. Nutritional Components in Early Lactation Cow Feeding.

Ingredient	Weight/Cow (kg)	DM 1 (%)	Final DMI 1 (kg)
Hay Dairy	2.50	88.50	2.21
Grass Silage	10.75	31.80	3.42
Oats	5.99	36.20	2.17
Barley-Dakota	11.50	40.00	4.80
Corn	13.52	31.50	4.26
Whey	2.75	17.00	0.47
Protein	4.75	93.30	4.43
Energy Dairy	4.25	88.00	3.74
Ground Barley	1.75	88.00	1.54
Mineral and Fat	1.26	97.26	1.23
Total	59.02	47.56	28.07

[1]Dry matter intake (DMI) is computed by multiplying the percentage of dry matter (DM%) in the given feed by the weight (kg) of the offered feed. The daily DMI goal is established at 2% of the cow's body weight.
